# The noncoding RNA LINC00152 conveys contradicting effects in different glioblastoma cells

**DOI:** 10.1038/s41598-021-97533-8

**Published:** 2021-09-16

**Authors:** Stefanie Binder, Ivonne Zipfel, Claudia Müller, Karolin Wiedemann, Carolin Schimmelpfennig, Gabriele Pfeifer, Kristin Reiche, Sunna Hauschildt, Jörg Lehmann, Ulrike Köhl, Friedemann Horn, Maik Friedrich

**Affiliations:** 1 Institute of Clinical Immunology, University of Leipzig, Leipzig, Germany; 2 Fraunhofer Institute for Cell Therapy and Immunology, Leipzig, Germany

**Keywords:** Molecular biology, Oncology

## Abstract

Glioblastoma multiforme (GBM) is an extremely aggressive brain tumor, characterized by its high genetic heterogeneity. In search of novel putative therapeutic RNA targets we investigated the role of the oncogenic long noncoding RNA LINC00152 (CYTOR, and STAiR18) in A172 glioblastoma cells. Here, we are the first to describe, that LINC00152 unexpectedly acts in a tumor suppressive manner in this cell line. SiRNA-based knockdown of LINC00152 enhanced malignant tumor behaviors including proliferation, cell cycle entry, migration, and invasion, contradicting previous studies using U87-MG and LN229 glioblastoma cells. Furthermore, LINC00152 knockdown had no influence on survival of A172 glioblastoma cells. In a genome wide transcription analysis of A172 and U87-MG glioblastoma cells, we identified 70 LINC00152 target genes involved in locomotion, cell migration, and motility in A172 cells, whereas in U87-MG cells only 40 target genes were detected. The LINC00152-regulated genes found in A172 differed from those identified in U87-MG glioblastoma cells, none of them being regulated in both cell lines. These findings underline the strong genetic heterogeneity of glioblastoma and point to a potential, yet unknown risk addressing LINC00152 lncRNA as a prospective therapeutic target in GBM.

## Introduction

Glioblastoma multiforme (GBM) is the most aggressive and most deadly type of brain cancer. It is a highly invasive and very heterogeneous tumor, which makes therapy approaches extremely difficult. The average survival after diagnosis is 12–15 month, with fewer than 5% of patients surviving longer than 5 years. Treatment includes surgery, radiotherapy, and temozolomide-based chemotherapy, but usually the cancer recurs^1^. New treatment strategies and novel therapeutic targets are urgently needed.

Long noncoding RNAs (lncRNAs) are often deregulated in cancer. Some of those lncRNAs were found to be overexpressed in tumors and to cause tumor progression (e.g. enhanced cell vitality, proliferation, migration, or invasion behavior)^2^. Hence, they represent a potential source of novel diagnostic and therapeutic targets^3^. New tools for cancer therapy include small interfering RNAs (siRNAs), a common form of RNAi-based therapeutics, silencing specific mRNAs and lncRNAs associated with cancer progression^4–6^. This new class of drugs targeting specific cancer-associated RNAs, i.e. ONPATTRO^®^ (patisiran)^7^ and GIVLAARI^®^ (givosiran)^8^, has recently been approved by the FDA, and in combination with traditional anticancer drugs, synergistic effects are anticipated^4,9,10^. Furthermore, most recently developed nanoparticle-based delivery systems give hope to major problems of RNA delivery, stability and intracellular release^4,9,11,12^.

In previous studies, we found the long noncoding RNA LINC00C152 (also known as CYTOR, and STAiR18) to be overexpressed in a wide range of different tumor entities, which makes it a promising target for clinical use. Furthermore, we carried out a detailed analysis of LINC00C152 in Multiple Myeloma (MM) cells, discovering an impressive survival phenotype^13,14^. The Knockdown of LINC00152 dramatically decreased cell vitality in myeloma cells^14^. We showed that LINC00152 specifically associates with the STAT3 pre-mRNA and that the knockdown of LINC00152 reduced the transcription factor STAT3 at both, the RNA and protein level, suggesting a positive feedback between both molecules. According to these data LINC00152 very likely can be regarded as an epigenetic modulator. Here we asked, whether the LINC00152 knockdown phenotype observed in MM could also be found in glioblastoma cells and whether LINC00152 is suitable as a putative therapeutic target in GBM.

Only recently it has been reported, that LINC00152 acts as a potent validated oncogene in glioblastoma cells and that it may serve as a therapeutic target for glioblastoma treatment^15–17^. The knockdown of LINC00152 in U87-MG and LN229 cells effectively suppressed malignant tumor behaviors including migration, invasion, proliferation, and epithelial–mesenchymal transition (EMT)^15,17^, whereas its overexpression supported tumor behaviors in U87-MG cells^16^.

Here, we uncovered an unexpected role of LINC00152 in A172 glioblastoma cells. It acts in a tumor suppressing manner and not as previously described as an oncogene^15–17^. The knockdown of LINC00152 in A172 cells resulted in an enhanced migration and invasion phenotype and furthermore, differences in target genes were observed compared to U87-MG glioblastoma cells. This is an important finding, demonstrating that the knockdown of apparently obvious therapeutic lncRNA targets in highly heterogeneous tumors like glioblastoma may achieve unwanted, tumor-stimulating effects in a subset of tumor cells.

## Results

### LIN00C152 displays different properties in myeloma and A172 glioblastoma cells

**Figure 1:**
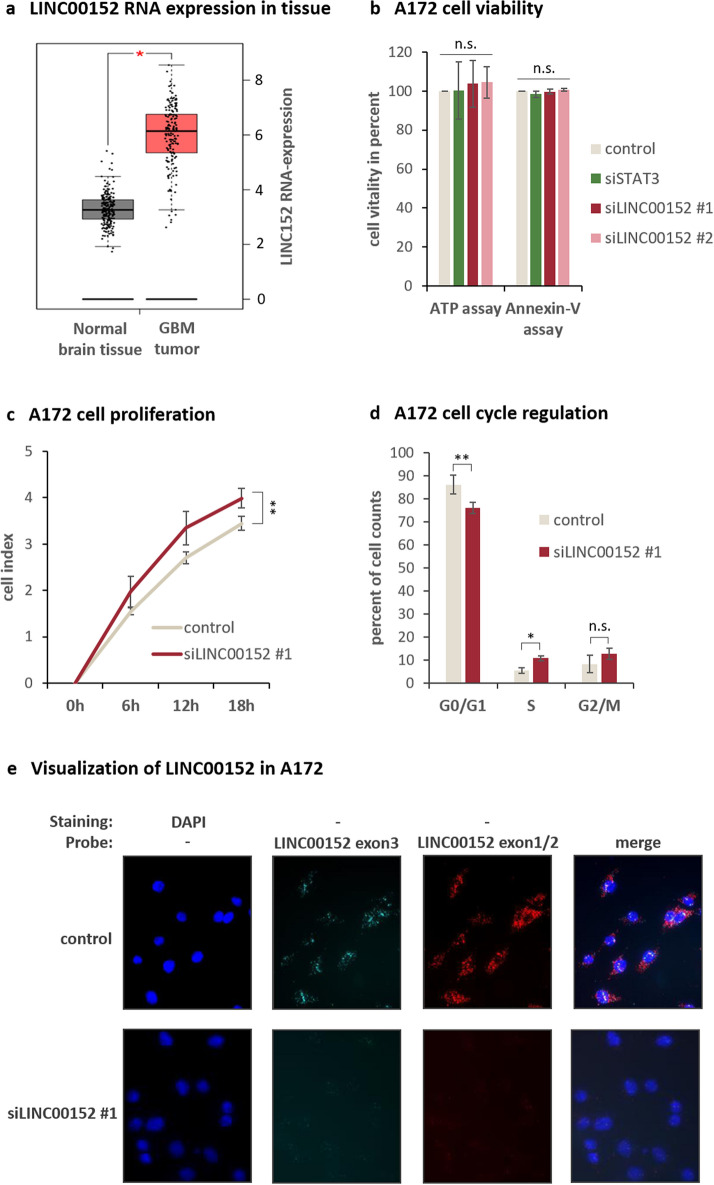
Characterization of LINC00152 functions in A172 glioblastoma cells. (**a**) GEPIA Plot displayed an increased LINC00152 expression in Glioblastoma Multiforme (GBM) tumor samples compared to healthy donor samples (normal brain tissue). (**b**) Cell viability was determined 48 h after LINC00152 and STAT3 knockdown using an ATP-based and an Annexin-V assay. Values were normalized to a siRNA negative control. (**c**) LINC00152 was knocked down and cell proliferation was monitored over time in an incubator equipped with a microscope and camera (XCelligence^®^). The impedance is recorded by RTCA software (https://www.agilent.com/en/product/cell-analysis/real-time-cell-analysis/rtca-software) and given as cell index, which is a measure of cell proliferation. (**d**) DNA of A172 cells was labled with propidium iodide 72 h after siLINC00152 #1 and control siRNA transfection and the DNA content was measured by flow cytometry. (**e**) Specific probes targeting LINC00152 either in exon3 (detecting primary and spliced transcript) or intron-spanning in exon 1 and -2 (detecting just spliced transcripts) were added to formaldehyde fixed A172 cells 48 h after knockdown with siLINC00152 #1 and a negative control siRNA. Nuclei were counterstained with DAPI and images were merged.

Initially we aimed to determine whether the knockdown of LINC00152 in glioblastoma (GBM) results in the same anti-tumor phenotype as it has been observed in myeloma cells^14^. Since LINC00152 was also found to be overexpressed in GBM (Fig. 1a), we supposed the same underlying molecular mechanisms. Therefore, we chose the A172 cell line (established from a glioblastoma of a 53-year-old man), which is routinely used as an in vitro model to test molecular therapies for glioblastoma^18^. Different siRNAs were used targeting LINC00152, which showed knockdown efficiencies of about 90 to 80 percent for siLINC00152 #1 and #2, respectively (Supplementary Fig. 1a). Whereas in myeloma cells, the siRNA-based knockdown of LINC00152 led to a strong decrease in cell viability and vitality, in A172 glioblastoma cells the knockdown had no effect (Fig. 1b). Unexpectedly, proliferation of A172 cells was increased (Fig. 1c), and an enhanced G0/G1- to S-phase cell cycle progression was detected after LINC00152 knockdown (Fig. 1d), which could be confirmed by siLINC00152 #2 (Supplementary Fig. 1b and c). Additionally, the localization of LINC00152 in A172 glioblastoma cells was visualized by ViewRNA™ analysis (Fig. 1e), using specific DNA probes recognizing the unspliced, nuclear transcript and mature RNA variants in the cytoplasm. The specificity of the method was verified by treating the cells with siRNAs, which led to a complete loss of signals. As a control for cellular localization, the nuclear located lncRNA MALAT-1 and the cytoplasmic mature mRNA of GAPDH were visualized using specific DNA probes (Supplementary Fig. 2).

In previous studies we observed in Multiple Myeloma a positive feedback between LINC00152 and the transcription factor STAT3 at the transcriptional level. STAT3 induced the expression of LINC00152 lncRNA and vice versa LINC00152 raised the expression of STAT3 mRNA by a physical interaction with the STAT3 pre-mRNA^14^. To test whether such a positive feedback circle also exists in GBM cells, we performed knockdown experiments targeting STAT3 and LINC00152 by siRNAs. The resulting expression levels indicate that LINC00152 positively influences STAT3 expression also in A172 glioblastoma cells (Supplementary Fig. 3a), by a direct interaction between LINC00152-RNA and STAT3-pre-mRNA (detected by ChIRP experiments; Supplementary Fig. 3b). However, in contrast to myeloma cells, the STAT3 transcription factor does not induce LINC00152 expression in A172 glioblastoma cells (Supplementary Fig. 3c).

### LINC00152 knockdown stimulates cell migration and invasion in A172 GBM cells

**Figure 2:**
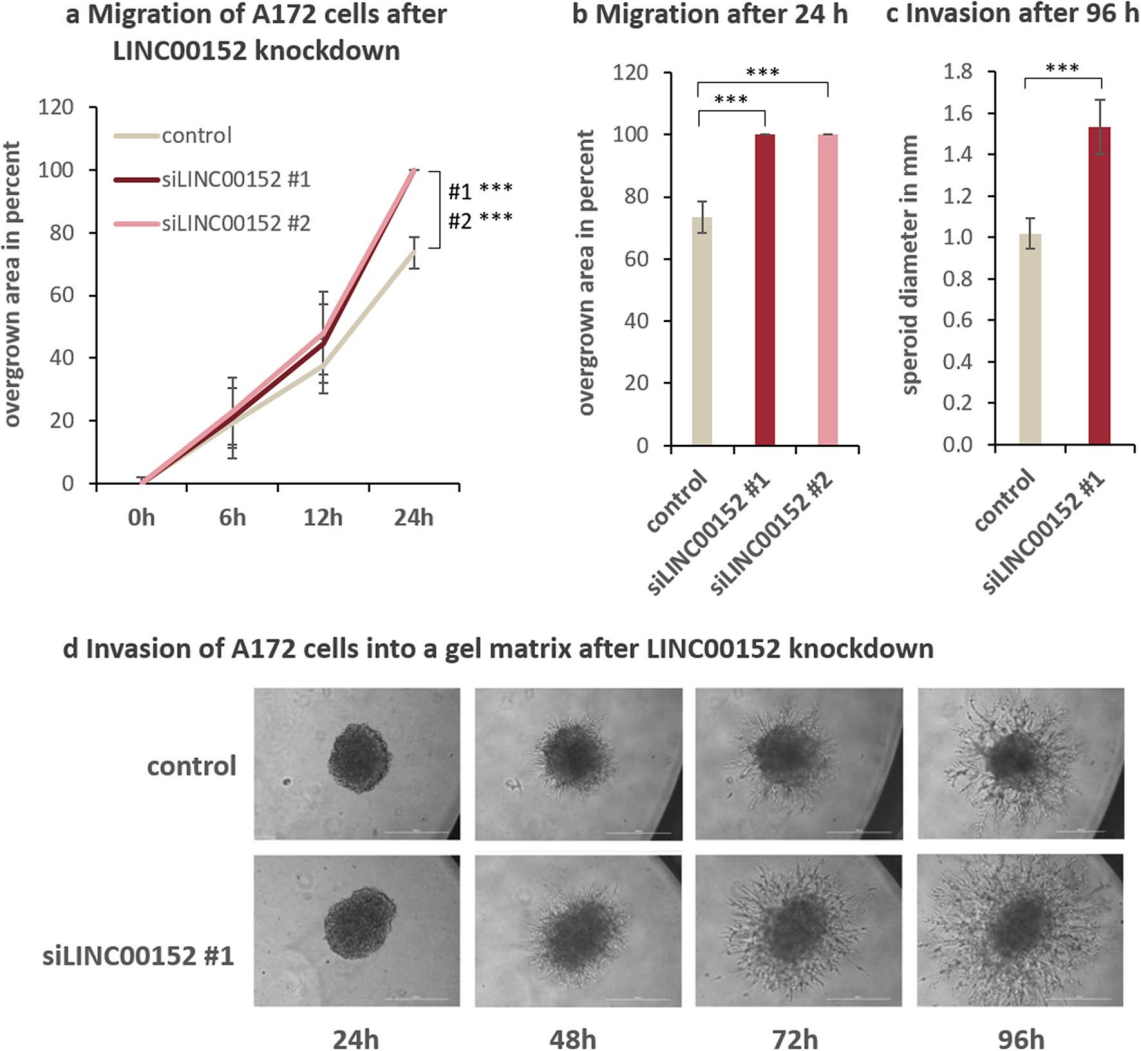
LINC00152 knockdown enhances migration and invasion of A172 GBM cells. (**a**) Migration of cells after LINC00152 knockdown through a cell-free gap of defined width was compared to cells transfected with a negative control siRNA over time (n = 4) and pictures were taken every 6 h. The images were analyzed using a Scratch Assay Analyzer (ImageJ https://imagej.nih.gov/ij/). (**b**) After 24 h the gap was completely overgrown by LINC00152 knockdown cells and up to 70% by control cells. (**c**,**d**) Invasion of LINC00152 knockdown cells into a gel matrix was compared to control siRNA-treated cells over time. Pictures were taken every 24 h (**d**) and after 96 h the spheroid diameter was measured and compared between LINC00152 knockdown and control cells in four independent biological replicates (**c**).

Assuming that a gene, which is overexpressed in Glioblastoma (refer to Fig. 1a) should have a tumor-promoting effect, we analyzed the influence of LINC00152 on tumor cell migration and invasion behavior. Therefore, an inlay with two-chambers separated by a dividing wall was inserted into cell culture dishes before cells were seeded. After cell attachment, the inlay was removed, creating a cell-free gap of a defined width. The cell-free gap was monitored while the cells migrated into the gap until it was completely covered. The migration assay was performed with A172 cells treated with LINC00152-siRNA or scramble-siRNA as a control. In contrast to our expectations, A172 cells lacking LINC00152 migrated faster than control cells (Fig. 2a,b). To exclude that the altered proliferation rates caused this effect, cells were pretreated with Mitomycin C. This compound inhibits proliferation whereas cell migration remains unimpaired. Similar results were obtained, confirming a migration phenotype (Supplementary Fig. 4). To analyze the invasiveness of A172 cells, a spheroid-forming assay was used. LINC00152 knockdown or control cells were cultured to form a spheroid, which was embedded in a gel matrix. The invasion of cells into the matrix gel was quantified. As seen in Fig. 2c,d the knockdown of LINC00152 caused an increased invasion of A172 cells. In conclusion, according to the cell migration and invasion behavior of A172 cells, LINC00152 does not display properties of a typical oncogene. On the contrary, LINC00152 seems to act as a tumor suppressor in A172 glioma cells.

### Gene expression pattern of A172- and U87-MG glioblastoma cells after LINC00152 knockdown

**Figure 3:**
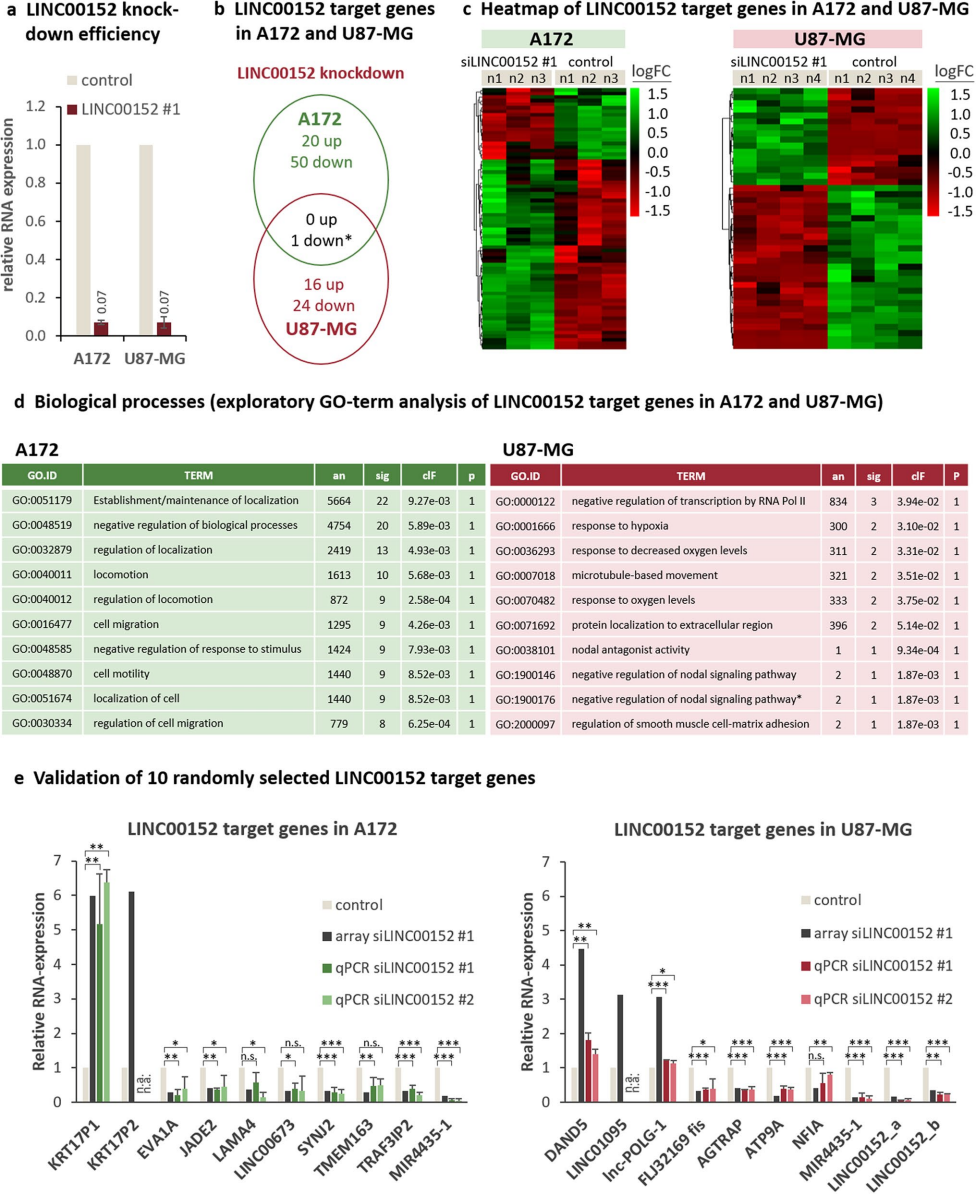
LINC00152 target genes in A172 and U87-MG glioblastoma cells. (**a**) Knockdown efficiencies of siLINC00152 #1 in A172 and U87-MG cells. RNA was prepared and reverse transcribed 48 h after transfection and knockdown efficiencies were determined by qPCR using specific primers. Values were normalized to U6 expression (housekeeper) and compared to the negative control knockdown. (b,**e**) For microarray analysis, knockdown of LINC00152 was performed in either A172 (n = 3) or U87-MG (n = 4) cells. 48 h after knockdown a microarray analysis was performed covering all human protein coding genes as well as several important noncoding transcripts. Differential gene expression of LINC00152 knockdown approaches was indicated compared to control knockdown. (**b**) Differences in LINC00152 target gene sets of A172 and U87-MG cells are shown in a Venn diagram. Only one LINC00152 target gene was found in both cell lines and corresponded to the down-regulated LINC00152 target gene itself, which was verified by three independent probes (red asterisk). (**b**) Heatmap of LINC00152 target genes in A172 and U87-MG cells. (**c**) Exploratory GO-term analysis of LINC00152 target genes in A172 and U87-MG. The top10 assigned biological processes are shown for both cell lines (an = annotated: number of genes in org.Hs.eg.db (Bioconductor) which are annotated with the GO-term; *sig* significant: number of genes in our dataset which are annotated with the GO-term; clF = classicFisher: p-value obtained after classic Fisher test; p = adjusted p-value: adjustment by Benjamini–Hochberg method). (**d**) 10 randomly selected LINC00152 target genes were validated by qPCR for both cell lines 40 h after LINC00152 knockdown. Values were normalized to U6 RNA (n = 4). The detected expression of equivalent genes identified by microarray is shown in black for comparison.

Before assessing the LINC00152-regulated gene sets between A172 and U87-MG cells, we showed comparable knockdown efficiencies in both cell lines (Fig. 3a). Afterwards, a genome-wide gene expression microarray was performed 48 h after LINC00152 knockdown in A172 and U87-MG. In total, 71 genes of A172 and 41 genes of U87-MG cells (log2 fold change > 0.5, and FDR < 0.2) were classified to be differentially regulated (Fig. 3b, Supplementary Table 1, Supplementary Table 2). Because of these low effects, analyses were made on discovery level. Thus, the sets of potentially regulated genes of both cell lines have to be validated, but it is noteworthy that they are completely different and only overlap in a single gene, the siRNA knockdown target LIN00152 itself (Fig. 3b). Most target genes were found to be down-regulated in both cell lines (50 down- to 20 up-regulated genes versus 24 down- to 16 up-regulated genes in A712 and U87-MG, respectively as shown in Fig. 3b,c). To identify biological processes in which these LINC00152 target genes are involved, we applied an exploratory Gene Ontology (GO) term enrichment analysis. In A172 glioblastoma cells, biological processes such as regulation of locomotion, cell migration, and motility were identified whereas in U87-MG cells negative regulation of transcription, responses to oxygen and microtubule-based movement dominated (Fig. 3d). To estimate the validity of the candidate gene sets and the associated results, a set of 10 randomly selected LINC00152 target genes per cell line was quantified by RTqPCR. As seen in Fig. 3e, nine out of ten target genes could be detected by qPCR and all of them showed the same regulation pattern as observed by the microarray analysis.

To identify the genomic differences between A172 and U87-MG, that might contribute to the contradictory phenotype, we compared mutant genes in both cell lines stored in the Harmonizome database^19^. A total number of 76 mutant genes were detected in each cell line. Among them, 29 genes are mutated in both lines while 47 genes were found specifically mutated in A172 or in U87-MG (Supplementary Fig. 5). To further assign A172 and U87-MG cells to one of the four molecular GBM subgroups (classical, mesenchymal, neural and proneural), we compared their gene expression patterns archived in the EMBL-EBI Expression Atlas with a predictive 840 gene signature described by Verhaak^20^. In U87-MG cells many marker genes of the mesenchymal subtype are highly expressed in contrast to A127. In A172 cells, we tend to find more genes of the proneural subtype upregulated (Supplementary Fig. 6). However, precise assignment is not possible.

We next wanted to investigate whether the expression patterns of A172 matched primary tumor samples (low-grade gliomas and glioblastomas). In these analyses, other glioblastoma cell lines (U87-MG, YKG1, and LN229) and healthy brain tissue were used for comparison. For this purpose, Principle Component Analysis was performed based on TCGA data of the complete gene expression patterns (Supplementary Fig. 7). All tumor cell lines show more similarities to the primary tumor samples (LGG in grey and GBM in dark blue), with a tendency to GBM than to healthy brain tissue. Based on the mRNA expression profile, we observed that among all cell lines the A172 cells cluster closest to some primary tumor samples. Moreover, PCA revealed an overlap with primary GBM tumor specimen (TCGA-08-0386-01) and two primary LLG tumor specimens (TCGA-DU-A7TJ-01 and TCGA-DU-6406-01). Note, these primary tumor specimens are located at the outer rim of the GBM and LGG tumor cloud. This indicates that they have genetic differences to the average of the primary tumors. It is an indication that patients might have tumor cells that resemble A172.

### The knockdown of LINC00152 in other glioblastoma cell lines leads to a tumor suppressive phenotype, suggesting a bona fide oncogenic function

**Figure 4:**
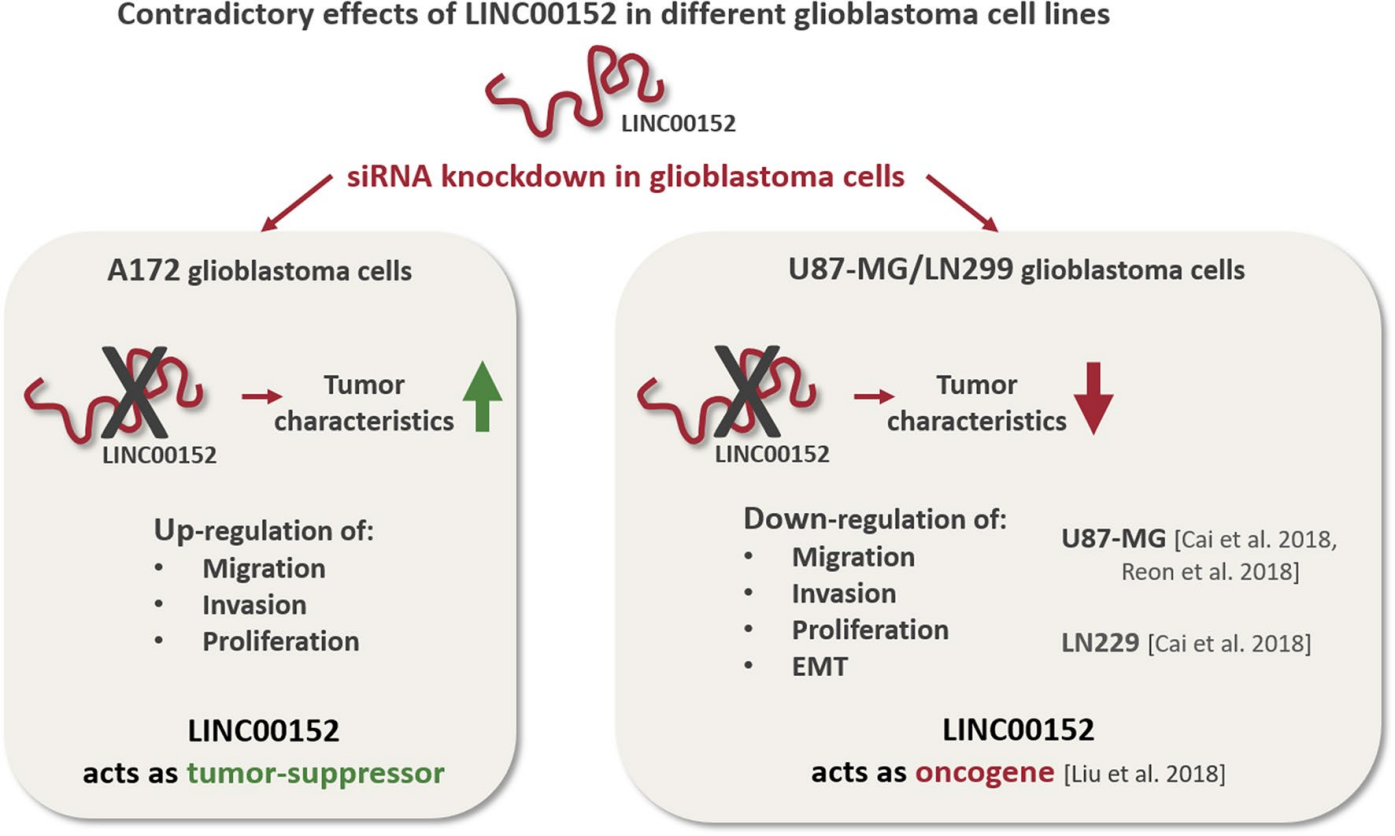
LINC00152 knockdown leads to contrary phenotypes in different glioblastoma cell lines. LINC00152 knockdown in U87-MG and LN229 glioblastoma cells has been shown to result in a decrease of cell migration, invasion, proliferation and EMT, indicating its oncogenic properties (right box). However, as we observed in A172 glioblastoma cells, LINC00152 seems to have opposing features (left box).

According to recent publications, LINC00152 acts as a potent oncogene in glioblastoma cells^15–17^. In contrast to our observations, these studies, which were carried out with U87-MG and LN229 cell lines, showed that LINC00152 knockdown effectively suppressed malignant tumor behaviors including migration, invasion, proliferation, EMT, and therefore, may serve as a therapeutic target for glioblastoma treatment (Fig. 4)^15,17^.

Since the methods used in those publications slightly differed from the ones described here, we tested whether the published phenotype in U87-MG cells can be confirmed in our hands. We compared the migration and cell cycle regulation of the two cell lines using an automated live-cell imaging system (IncuCyte^®^). As seen in Supplementary Fig. 8, we confirmed both, the published migration inhibiting phenotype of the LINC00152 knockdown in U87-MG cells associated with a G0/G1 cell cycle arrest as well as the opposite effect in A172 glioblastoma cells.

To exclude a cellular contamination or the use of an incorrect cell line, short tandem repeat analysis and profiling of the cells was performed by the company CLS GmbH (Eppelheim, Germany). The cell lines were clearly identified as A172 and U87-MG, respectively (Supplementary Fig. 9).

## Discussion

The long noncoding RNA (lncRNA) LINC00152 is overexpressed in various different cancer entities and its high expression is associated with a poor prognosis in most of them^17^. The ability to promote tumor progression designates LINC00152 as a valid oncogene and promising therapeutic target^14,21^. LINC00152 is also overexpressed in glioblastoma, a highly aggressive brain tumor with a very poor prognosis, for which novel treatment methods are urgently needed^22^. Whereas previous studies have shown that LINC00152 is an oncogenic lncRNA promoting invasion, migration, proliferation and EMT in gliomas^15–17^, here for the first time we describe an unexpected role of the human LINC00152 as a tumor suppressor in A172 glioblastoma cells.

The initial aim of the study was to test, whether the tumor-promoting activity of LINC00152 described in multiple myeloma cells (MM) is also seen in glioblastoma^14^. As a cellular model we used A172, a common glioblastoma cell line that is also frequently used in current drug trials^23–28^. While the knockdown of LINC00152 dramatically decreased the survival of myeloma cells^14^, no effect on viability was found in A172 cells. Although LINC00152 interacts with the transcription factor STAT3 mRNAs in both, myeloma and A172 glioblastoma cells, STAT3 only regulates LINC00152 expression in myeloma but not glioblastoma cells. This difference could be explained by the fact, that survival of myeloma cells strictly depends on continuous Interleukin-6 (IL-6) supply and thus on the activation of the STAT3 signaling pathway^29,30^. This is not the case for glioblastoma cells.

Further analysis revealed an enhancement of both, tumor cell migration and invasion behavior after knocking down LINC00152 in A172 cells, suggesting an unexpected role of LINC00152 as a tumor suppressor. These findings are in contrast to at least 3 other recent publications, describing LINC00152 as a well-defined oncogene in other glioblastoma cells (U87-MG, LN229)^15–17^. By using the here described experimental setting, we could confirm the oncogenic function of LINC00152 in U87-MG cells, a cell line mainly used in those publications.

The mechanism underlying the action of LINC00152 is not exactly known yet. There are conflicting reports on exactly how LINC00152 promotes the invasive phenotype in glioblastoma. Several studies have suggested that LINC00152 acts as a competing endogenous RNA (ceRNA) by depleting microRNAs, e.g. miR-103a-3p (FEZF1/CDC25A pathway)^31^, miR-107^16^, and miR-612 (AKT2/NF-κB pathway)^15^. In contrast, the most recent report rules out a mechanism as a miRNA sponge and instead suggests a direct interaction between a LINC00152 3´-hairpin structure and distinct proteins^17^.

In a genome wide transcription analysis of A172 cells, we identified 70 LINC00152 target genes involved in locomotion, cell migration, and motility. In U87-MG glioblastoma cells we found 40 regulated target genes involved in negative regulation of transcription and response to oxygen. The finding that none of the genes do overlap might underline the strong genetic heterogeneity of glioblastoma cells.

Both A172 and U87-MG cell lines sustain the main features of glioblastomas and therefore can serve as tools when investigating this kind of neoplasms^32,33^. GBM is a very heterogenous tumor that can be separated into different subtypes e.g. mesenchymal, classical, proneural and neural, based on their transcriptional profile^17,20,34^. The assignment of the cell lines to one of these GBM subtypes according to a signature of 840 molecular subgroup defining genes revealed a rather mesenchymal genotype for the U87-MG and a rather proneural genotype for the A172 cells. Moreover, we were able to show that among all glioblastoma cell lines tested, A172 cells cluster closest to some primary tumor samples. The identity of the A172 und U87-MG cell lines used here was confirmed by short tandem repeat profiling authentication.

Since new RNAi based therapies are now entering the clinic^7,8^, therapeutic targets based on oncogenic lncRNAs are moving strongly into the medical research focus^10^. Our findings that oncogenic LINC00152 acts as a tumor suppressor in A172 glioblastoma cells point out to a potential yet unknown risk, if lncRNAs were addressed as targets in very heterogeneous tumors. LncRNAs were shown to have diverse regulatory functions and to act in guiding the fine-tuning of gene regulation in a cell specific manner. This could render some lncRNAs particularly susceptible to contradictory functions in different genetic constellations.

In this study, we are the first to describe an unexpected role of the oncogenic acting LINC00152 as a tumor suppressor in A172 GBM cells. Thereby, the study has the following limitations: Experiments were carried out on two GBM cell lines (A172 and U87-MG) only. In the context of establishing monolayer cultures, adaptation processes such as clonal selection, gene expression alterations, and genetic drift cannot be excluded. The experiments were only performed in vitro, since the A172 cell line cannot be engrafted in mice. Therefore, the eligibility of LINC00152 as putative therapeutic target should not be abandoned in general, based on our findings. However, our work should draw attention to this particular phenotype in future therapeutic investigations of this highly heterogeneous tumor disease.

Here, we present evidence, that the knockdown of an apparently obvious oncogenic lncRNA target in a highly heterogeneous tumor like GMB could even stimulate tumor properties and progression in a subsets of tumor cells.

## Methods

### Cell culture and RNAi-mediated knockdown

Human glioblastoma cell lines A172 and U87-MG were obtained by ATCC. The identity of both cell lines was analyzed and verified by the company Cell Line Service GmbH (Eppelheim, Germany) and cell authentications are shown in the Supplement (see Supplementary Fig. 1). Cells were maintained in DMEM containing GlutaMAX™ (LIFE Technologies, Carlsbad, California, USA), supplemented with 10% fetal calf serum (Lonza, Basel, Switzerland) and 1% penicillin/streptomycin (LIFE Technologies). Transfection was conducted with either 200 pmol stealth-siRNAs (Thermo Fisher, Waltham, Massachusetts, USA; Supplementary Table 3) or a medium GC control (Invitrogen, Carlsbad, California, USA) per 5 × 10^6^ cells. Transfection was carried out using the NEON™-Kit and the microporator MP100 Digitalbio (LIFE Technologies) according to the manufacturer’s instructions. One pulse of 1300 V and 30 ms was applied. Knockdown cells were cultivated 24 to 48 h post transfection as indicated for every experiment.

### Cell vitality assays

Apoptosis rates were determined by the Dead Cell Apoptosis Kit (LIFE Technologies) according to the manufacturerˈs protocol. Cell vitality was examined by CellTiter-Glo® Luminescent Cell Viability Assay (Promega, Madison, Wisconsin, USA). Analysis of apoptotic cells was carried out by FACSCalibur™ (BD Biosciences, Franklin Lakes, New Jersey, USA) together with the corresponding software BD CellQuest™ Pro (https://www.bd.com/en-uk/products/molecular-diagnostics/cytometric-analysis-products) and cell vitality was detected using LUMIstar Optima (BMG Labtech, Ortenberg, Germany).

### Cell cycle analysis

For cell cycle analysis, 5 × 10^5^ cells were fixed 72 h after transfection in 4 mL ice cold ethanol (80%) for 20 min at − 20 °C. DNA content was labelled with propidium iodide (PI) by adding 100 µL PBS, 100 µL FACS buffer (0.1 M sodium acetate, 0.5 M EDTA), 50 µL RNase (Qiagen, 1 mg/mL FACS buffer), 20 µL Triton-X 100 (0.2% in FACS buffer) and 10 µL propidium iodide (1 mg/mL) for 30 min at 37 °C and measured in the FACSCalibur flow cytometer (BD Biosciences). Data represent the mean ± s.d. of a minimum of three biological replicates.

### Proliferation assay

Cell proliferation was analyzed using the xCELLigence^®^ instrument (ACEA Biosciences Inc., San Diego, California, USA). After transfection, 5 × 10^4^ cells were seeded in culture plates with microelectrodes (E-Plate^®^, ACEA Biosciences Inc.) followed by an impedance measurement over 50 h. The impedance is recorded by RTCA software (https://www.agilent.com/en/product/cell-analysis/real-time-cell-analysis/rtca-software) and given as cell index, which allows to draw conclusions about cell proliferation, morphology, and adherence.

### Cell migration assay

After transfection, 1 × 10^4^ cells per chamber were seeded into a two-chamber Idibi-insert. The insert was removed after 24 h generating a defined cell-free area of 500 µm width into which the cells migrate. The migration of cells with and without a 3-h pre-incubation of the proliferation-inhibitor 50 µg/ml mitomycinC (Roche, Basel, Switzerland) was monitored via microscopy every six hours and pictures were analyzed using the software ImageJ (https://imagej.nih.gov/ij/).

### Invasion assay

A 96-well plate with U-shaped wells was coated with a 1.5% agarose solution and 3 × 10^3 transfected cells were seeded per well and covered with culture medium. Within 24 h a spheroid was formed and transferred into an invasion matrix consisting of Geltrex™ and culture medium (1:1). The ingrowth of the spheroid into the invasion matrix was assessed microscopically over 72 h by determining the spheroid diameter.

### Fluorescence in situ hybridization

The ViewRNA™ (Thermo Fisher Scientific) staining was performed using the ViewRNA™ ISH Cell Assay Kit according to the manufacturer's instructions. The A172 cells were first fixed on poly-L-lysine coated cover tubes for 20 min with 2% formaldehyde with the addition of 0.8% acetic acid and then protease digested (1:4000) to ensure accessibility for the probes even in the nucleus of the cells. The probes (listed in Supplementary Table 4) were then hybridized against the different isoforms of LINC00152. Positive controls were probes against the cytoplasmic GAPDH mRNA and the nuclear ncRNA MALAT-1. Probes against the bacterial RNAs of lacZ and dapB served as negative controls. To further verify the specificity of the signals, LINC00152 was also stained in A172 cells that had previously been subjected to RNAi-mediated STAiR18-knockdown. After in situ hybridization, the nuclei were counterstained with DAPI. The fluorescence signals were analyzed microscopically using FITC (488 nm), Cy3 (550 nm) and Cy5 (650 nm) filters.

### RNA isolation and RTqPCR analysis

RNA was isolated with TRIzol (LIFE Technologies) following the manufacturerˈs protocol. RNA was DNase-digested using TURBO-DNA-free Kit (LIFE Technologies). Reverse transcription of RNA was conducted using the RevertAid First Strand cDNA synthesis kit (Thermo, Waltham, Massachusetts, USA). QPCR-analyses of cDNA were performed using SYBR Green MasterMix (BIO-Rad, Hercules, California, USA) as described by the manufacturer using specific primers (listed in Supplementary Table 5) and the CFX Real time PCR detection system (Bio-Rad, Hercules, California, USA).

### Global gene expression analysis

Gene expression analyses were performed in A172 and U87-MG cells 48 h after LINC00152 knockdown in three (A172) or four (U87-MG) independent biological replicates each. Samples were prepared using SurePrint G3 Human Gene Expression v2 8 × 60 K Microarrays (Agilent Technologies, Santa Clara, USA, California) and the belonging OneColor Quick Amp Labeling kit according to the manufacturer’s instructions. Quality controlled libraries were hybridized to the array and signal detection occurred by microarray scanner (Agilent Technologies). Raw data files were processed by R v4.0.0 and Bioconductor package limma v3.46.0. Raw data files were analyzed as described in Supplementary Methods. Data were deposited in the GEO database [GSE161352].

## Supplementary Information

Supplementary Information.

## Data Availability

The data sets supporting the results of this article are available in the GEO database repository [GSE161352].
