# Supplementary material for: The noncoding RNA LINC00152 conveys contradicting effects in different glioblastoma cells
Source: Sci Rep. 2021 Sep 16;11:18499. doi: 10.1038/s41598-021-97533-8 (PMC8446032; doi:10.1038/s41598-021-97533-8)
Supplement: Supplementary file 1 — Supplementary Information. [file 41598_2021_97533_MOESM1_ESM.docx]

Supplementary Information

# **The noncoding RNA LINC00152 conveys contradicting effects in different glioblastoma cells**

Stefanie Binder^1 +*^, Ivonne Zipfel^1 +^, Claudia Müller^3^, Karolin Wiedemann^2^, Carolin Schimmelpfennig^2^, Gabriele Pfeifer^1^, Kristin Reiche^2^, Sunna Hauschildt^1,4^, Jörg Lehmann^2^, Ulrike Köhl^1,2,3^, Friedemann Horn^1,2§^ and Maik Friedrich^1§^

^1^ Institute of Clinical Immunology, Faculty of Medicine, University of Leipzig, Germany

^2^ Fraunhofer Institute for Cell Therapy and Immunology, Department of Diagnostics, Leipzig, Germany

^3^ Fraunhofer Institute for Cell Therapy and Immunology, Department of Therapy Validation, Leipzig, Germany

^4^ Institute of Biology, Division of Cell & Developmental Biology, University of Leipzig, Germany

^+^ both authors share first position

^§^ both authors share last position

^*^ is the corresponding author

Stefanie Binder stefanie.binder@medizin.uni-leipzig.de

Ivonne Zipfel zipfel.ivonne@uni-leipzig.de

Claudia Müller claudia.mueller@izi.fraunhofer.de

Karolin Wiedemann karolin.wiedemann@izi.fraunhofer.de

Carolin Schimmelpfennig carolin.schimmelpfennig@izi.fraunhofer.de

Gabriele Pfeifer gabriele.pfeifer@medizin.uni-leipzig.de

Kristin Reiche kristin.reiche@izi.fraunhofer.de

Sunna Hauschildt shaus@rz.uni-leipzig.de

Jörg Lehmann joerg.lehmann@izi.fraunhofer.de

Ulrike Köhl ulrike.koehl@izi.fraunhofer.de

Friedemann Horn friedemann.horn@izi.fraunhofer.de

Maik Friedrich maik.friedrich@medizin.uni-leipzig.de

# **Supplementary Methods**

## **Scratch wound migration assay (IncuCyte®)**

Cells were transfected and 1x10^4 cells per well were seeded into a 96 well IncuCyte® plate (Sartorius, Göttingen, Germany) as quadruplicates. 24h post-transfection a defined scratch was applied per well using WoundMaker (Sartorius) and the plate was placed into the IncuCyte® incubator with integrated microscope (Sartorius) for three days. Pictures were taken automatically every two hours. Pictures were analyzed by the according IncuCyte® Base Analysis Software (<https://www.essenbioscience.com/de/products/software/incucyte-base-software/>) and values were normalized to time point 0 h. A minimum of three independent biological replicates, as technical quadruplicates each, were summarized as mean values with standard deviation.

## **Analyzation of microarray data**

Raw data files were processed by R v4.0.0 [R Core Team, 2020] and Bioconductor package limma v3.46.0 [Ritchie et al., 2015]. Quality control, normalization, including nonspecific filtering, the detection of expression variation was performed as previously published in TAPIR [Friedrich et al., 2020]. Because of low effects the differential gene expression analysis was made on discovery level. 71 (A712) and 41 (U87-MG) genes could be extracted with a log2 fold change > 0.5 and a FDR < 0.2. To find the intersection of up- and down-regulated genes, respectively, between the cell lines A172 and U87-MG limma::decideTest (method = “separate”) was used. Functional analysis among all significantly regulated genes compared to the background set of all genes covered by at least one probe of the microarray was conducted for gene ontology (GO) categories. Gene ontology enrichment analyses were realized by using the Bioconductor package topGO v2.42.0 [Alexa and Rahnenfuhrer, 2020]. To detect enriched GO categories, we applied Fisher’s exact test using the classic algorithm. P-values were adjusted for multiple testing (Benjamini-Hochberg correction).

## **Chromatin Isolation by RNA Purification (ChIRP)**

Chromatin isolation by RNA purification (ChIRP) was conducted according to Chu *et al*.2012. In short, 3′-biotinylated oligonucleotides (listed in the Supplementary Table 6) were designed complementary to the target ncRNA LINC00152 and to bacterial lacZ RNA as a negative control. 2 × 10^7^ cells per pulldown were fixed using 1% formaldehyde for 10 min and sonicated for 20 min using Bioruptor® (Diagenode, Seraing, Belgium). Pulldown was done by adding 100 pmol oligo mixture (12 oligos targeting LINC00152 in different positions) per 1 ml cell lysate, followed by an immobilization to streptavidin T1 magnetic Dynabeads® (Thermo). RNA pulldown fractions were analyzed by qPCR.

## **Immunoblot**

Proteins were isolated 48h after siRNA transfection using cell lysis buffer (50mM Tris/HCl pH 7.2, 150mM NaCl, 5mM NaF, 0.25mM EDTA, 1% Triton-X-100, 1% SDS, 1mM NaVO4, 5μg/ml pepstatin, 5μg/ml leupeptin, 0.14U/ml aprotinin). After a Bradford analysis, 30μg of lysates were supplemented with Laemmli buffer, boiled, separated in a 10% SDS gel and blotted to a polyvinylidene fluoride membrane. Protein bands were visualized using primary and secondary antibodies (listed in Supplementary Table 7), the Super Signal® West Dura detection reagent (Thermo), and a CCD camera (Raytest, Straubenhardt, Germany). Each immunoblot shown is a representative example out of a minimum of two independent biological replicates (n > 2).

# **Supplementary Tables**

## **Supplementary Table 1**

**Supplementary Table 1: LINC00152 target genes in A172 cells.**

| **ProbeName** | **SystematicName** | **GeneSymbol Agilent** | **logFC** | **adj.P.Val** | **P.Value** |
| --- | --- | --- | --- | --- | --- |
| A_24_P273143 | NR_024204 | LINC00152 | -2,68 | 0,005 | 0,000 |
| A_23_P139786 | NM_003733 | OASL | -2,56 | 0,009 | 0,000 |
| A_21_P0012079 | NR_024373 | MIR4435-1HG | -2,49 | 0,012 | 0,000 |
| A_24_P123408 | NM_014945 | ABLIM3 | -2,40 | 0,009 | 0,000 |
| A_23_P252052 | NM_182909 | FILIP1L | -2,39 | 0,168 | 0,000 |
| A_24_P265832 | NM_033050 | SUCNR1 | -2,27 | 0,031 | 0,000 |
| A_23_P207507 | NM_003786 | ABCC3 | -2,19 | 0,031 | 0,000 |
| A_24_P216456 | NM_001004343 | MAP1LC3C | -2,18 | 0,128 | 0,000 |
| A_23_P46844 | NM_030912 | TRIM8 | -2,13 | 0,031 | 0,000 |
| A_33_P3571901 | XM_002342310 | LOC100287387 | -2,09 | 0,058 | 0,000 |
| A_23_P407206 | NM_018941 | CLN8 | -1,95 | 0,044 | 0,000 |
| A_23_P210176 | NM_000210 | ITGA6 | -1,89 | 0,069 | 0,000 |
| A_24_P93664 | NR_073154 | C1orf145 | -1,88 | 0,031 | 0,000 |
| A_24_P364807 | NM_017839 | LPCAT2 | -1,88 | 0,031 | 0,000 |
| A_33_P3341656 | NM_005920 | MEF2D | -1,85 | 0,131 | 0,000 |
| A_33_P3289025 | NM_001135032 | EVA1A | -1,83 | 0,083 | 0,000 |
| A_21_P0011334 | ENST00000558082 | - | -1,83 | 0,136 | 0,000 |
| A_32_P138042 | BX445743 |  | -1,83 | 0,031 | 0,000 |
| A_23_P39550 | NM_030923 | TMEM163 | -1,82 | 0,061 | 0,000 |
| A_21_P0011675 | TCONS_l2_00012321 | - | -1,76 | 0,053 | 0,000 |
| A_21_P0005963 | XR_110877 | - | -1,71 | 0,031 | 0,000 |
| A_19_P00319528 | ENST00000522718 | - | -1,70 | 0,048 | 0,000 |
| A_32_P743407 | NM_032569 | GLYR1 | -1,64 | 0,046 | 0,000 |
| A_19_P00319646 | NR_036488 | LINC00673 | -1,62 | 0,083 | 0,000 |
| A_24_P413126 | NM_020182 | PMEPA1 | -1,61 | 0,145 | 0,000 |
| A_23_P206280 | NM_201525 | GPR56 | -1,60 | 0,151 | 0,000 |
| A_33_P3298024 | NM_001144070 | ABCC3 | -1,57 | 0,053 | 0,000 |
| A_23_P110879 | NM_147686 | TRAF3IP2 | -1,56 | 0,053 | 0,000 |
| A_24_P294821 | NM_003898 | SYNJ2 | -1,56 | 0,083 | 0,000 |
| A_23_P302005 | NM_006873 | STON1 | -1,56 | 0,053 | 0,000 |
| A_21_P0001676 | TCONS_00002016 | - | -1,53 | 0,031 | 0,000 |
| A_23_P64129 | NM_006410 | HTATIP2 | -1,52 | 0,037 | 0,000 |
| A_24_P365365 | NM_003200 | TCF3 | -1,51 | 0,087 | 0,000 |
| A_23_P154065 | NM_006000 | TUBA4A | -1,51 | 0,053 | 0,000 |
| A_19_P00321068 | NR_003491 | MIAT | -1,50 | 0,127 | 0,000 |
| A_24_P111134 | NM_013382 | POMT2 | -1,46 | 0,168 | 0,000 |
| A_32_P40288 | NM_052913 | TMEM200A | -1,46 | 0,048 | 0,000 |
| A_24_P40907 | NM_203453 | PPAPDC2 | -1,44 | 0,145 | 0,000 |
| A_23_P151150 | NM_202002 | FOXM1 | -1,42 | 0,089 | 0,000 |
| A_21_P0001634 | TCONS_00001843 | - | -1,42 | 0,053 | 0,000 |
| A_33_P3341299 | NM_014405 | CACNG4 | -1,40 | 0,053 | 0,000 |
| A_33_P3308749 | NM_001105207 | LAMA4 | -1,40 | 0,131 | 0,000 |
| A_19_P00321124 | ENST00000435813 | - | -1,37 | 0,077 | 0,000 |
| A_33_P3266419 | NM_016433 | GLTP | -1,36 | 0,132 | 0,000 |
| A_21_P0001231 | ENST00000437416 | - | -1,31 | 0,099 | 0,000 |
| A_33_P3353692 | NM_002473 | MYH9 | -1,30 | 0,120 | 0,000 |
| A_33_P3323803 | NM_014644 | PDE4DIP | -1,29 | 0,112 | 0,000 |
| A_24_P226278 | NM_015288 | JADE2 | -1,29 | 0,069 | 0,000 |
| A_23_P143120 | NM_003183 | ADAM17 | -1,29 | 0,145 | 0,000 |
| A_21_P0009405 | ENST00000525111 | - | -1,25 | 0,084 | 0,000 |
| A_21_P0012077 | ENST00000409139 |  | -1,19 | 0,103 | 0,000 |
| A_33_P3286066 | NM_001134438 | PHLDB2 | -1,15 | 0,103 | 0,000 |
| A_21_P0010324 | TCONS_00029307 | - | -1,10 | 0,132 | 0,000 |
| A_23_P429950 | NM_000216 | KAL1 | 1,32 | 0,199 | 0,000 |
| A_33_P3255829 | NM_015419 | MXRA5 | 1,33 | 0,163 | 0,000 |
| A_21_P0012822 | NM_001164440 | ANKRD33B | 1,44 | 0,120 | 0,000 |
| A_23_P202104 | NM_005729 | PPIF | 1,50 | 0,069 | 0,000 |
| A_23_P74299 | NM_000674 | ADORA1 | 1,52 | 0,114 | 0,000 |
| A_33_P3261957 | NM_005795 | CALCRL | 1,62 | 0,111 | 0,000 |
| A_23_P36972 | NM_007129 | ZIC2 | 1,67 | 0,031 | 0,000 |
| A_23_P77545 | NM_001053 | SSTR5 | 1,68 | 0,070 | 0,000 |
| A_23_P258136 | NM_015419 | MXRA5 | 1,75 | 0,029 | 0,000 |
| A_23_P92909 | NM_205841 | SPINK6 | 1,83 | 0,132 | 0,000 |
| A_23_P94571 | NM_004432 | ELAVL2 | 1,90 | 0,053 | 0,000 |
| A_23_P383009 | NM_000599 | IGFBP5 | 1,98 | 0,009 | 0,000 |
| A_33_P3857239 | NR_033415 | KRT42P | 2,54 | 0,111 | 0,000 |
| A_21_P0011578 | ENST00000577449 |  | 2,58 | 0,096 | 0,000 |
| A_24_P882732 | ENST00000582047 | - | 2,61 | 0,024 | 0,000 |
| A_24_P887857 | A_24_P887857 | - | 2,77 | 0,079 | 0,000 |
| A_33_P3345534 | NM_000526 | KRT14 | 3,24 | 0,049 | 0,000 |
| A_23_P38537 | NM_005557 | KRT16 | 3,30 | 0,089 | 0,000 |
| A_21_P0011517 | NM_000526 | KRT14 | 3,37 | 0,200 | 0,000 |
| A_32_P62963 | NR_029392 | KRT16P2 | 3,69 | 0,040 | 0,000 |

## **Supplementary Table 2**

**Supplementary Table 2: LINC00152 target genes in U87-MG cells.**

| **ProbeName** | **SystematicName** | **GeneSymbol Agilent** | **logFC** | **adj.P.Val** | **P.Value** |
| --- | --- | --- | --- | --- | --- |
| A_21_P0004156 | ENST00000507491 |  | -2,90 | 0,151 | 0,000 |
| A_21_P0012079 | NR_024373 | MIR4435-1HG | -2,81 | 0,002 | 0,000 |
| A_21_P0010671 | NR_038845 | LYPLAL1-AS1 | -2,61 | 0,089 | 0,000 |
| A_24_P273143 | NR_024204 | LINC00152 | -2,57 | 0,002 | 0,000 |
| A_23_P380614 | NM_006045 | ATP9A | -2,49 | 0,024 | 0,000 |
| A_21_P0001646 | TCONS_00001887 | - | -2,47 | 0,010 | 0,000 |
| A_33_P3375314 | NM_006045 | ATP9A | -2,46 | 0,021 | 0,000 |
| A_21_P0004600 | ENST00000569865 |  | -2,38 | 0,003 | 0,000 |
| A_21_P0003610 | TCONS_00008078 | - | -2,05 | 0,188 | 0,000 |
| A_21_P0004560 | TCONS_00010957 | - | -2,00 | 0,024 | 0,000 |
| A_21_P0012078 | NR_015395 | MIR4435-1HG | -1,95 | 0,090 | 0,000 |
| A_33_P3293698 | A_33_P3293698 | - | -1,95 | 0,188 | 0,000 |
| A_33_P3402694 | NM_014980 | STXBP5L | -1,76 | 0,080 | 0,000 |
| A_23_P45955 | NM_014466 | TEKT2 | -1,73 | 0,100 | 0,000 |
| A_33_P3847514 | NM_001145652 | C6orf141 | -1,71 | 0,157 | 0,000 |
| A_21_P0001218 | ENST00000424696 |  | -1,70 | 0,035 | 0,000 |
| A_33_P3350553 | ENST00000376964 | UXT | -1,69 | 0,080 | 0,000 |
| A_19_P00326132 | NR_003255 | TSIX | -1,63 | 0,111 | 0,000 |
| A_24_P709377 | NR_015377 | PAX8-AS1 | -1,60 | 0,092 | 0,000 |
| A_33_P3217317 | AK056731 |  | -1,59 | 0,162 | 0,000 |
| A_33_P3240295 | A_33_P3240295 | - | -1,51 | 0,157 | 0,000 |
| A_21_P0007395 | TCONS_00019752 | - | -1,50 | 0,151 | 0,000 |
| A_21_P0012077 | ENST00000409139 |  | -1,50 | 0,024 | 0,000 |
| A_21_P0011165 | TCONS_l2_00007037 | XLOC_l2_003877 | -1,48 | 0,089 | 0,000 |
| A_21_P0011950 | ENST00000451884 |  | -1,40 | 0,084 | 0,000 |
| A_33_P3319625 | NM_001134673 | NFIA | -1,29 | 0,089 | 0,000 |
| A_23_P158596 | NM_001040196 | AGTRAP | -1,28 | 0,151 | 0,000 |
| A_33_P3351745 | NM_024070 | PVRIG | 1,39 | 0,089 | 0,000 |
| A_24_P376379 | NR_033944 | LOC647323 | 1,40 | 0,151 | 0,000 |
| A_33_P3306146 | NM_001145031 | PLAU | 1,47 | 0,151 | 0,000 |
| A_33_P3300312 | NM_007329 | DMBT1 | 1,54 | 0,151 | 0,000 |
| A_33_P3355618 | NR_033370 | LOC63930 | 1,54 | 0,151 | 0,000 |
| A_21_P0007364 | TCONS_00019671 | - | 1,55 | 0,151 | 0,000 |
| A_33_P3326989 | NM_014226 | MOK | 1,60 | 0,024 | 0,000 |
| A_33_P3464749 | NR_045123 | LINGO1-AS1 | 1,61 | 0,057 | 0,000 |
| A_21_P0008036 | TCONS_00021729 | - | 1,61 | 0,097 | 0,000 |
| A_21_P0008777 | TCONS_00023778 | - | 1,62 | 0,089 | 0,000 |
| A_23_P50697 | NM_006905 | PSG1 | 1,63 | 0,080 | 0,000 |
| A_21_P0012806 | ENST00000505848 | LINC01095 | 1,64 | 0,090 | 0,000 |
| A_32_P209960 | NM_000246 | CIITA | 2,03 | 0,041 | 0,000 |
| A_23_P435636 | NM_152654 | DAND5 | 2,16 | 0,089 | 0,000 |
| A_23_P27128 | NM_152467 | KLHL10 | 2,23 | 0,005 | 0,000 |
| A_23_P303155 | NM_032824 | TMEM87B | 2,31 | 0,188 | 0,000 |

## **Supplementary Table 3**

**Supplementary Table 3: Stealth-siRNAs used for transfection.** Stealth-siRNAs were designed using BLOCK-iT RNAi Designer (Thermo Fisher Scientific) and were all already published in Binder et al. 2020.

| **Target** | **Sequence 5´-3´** | **Target region** |
| --- | --- | --- |
| siLINC00152 #1 | 5'-TGCCTGTCTTCAGATCTTCACAGCA-3' | Exon 1 |
| siLINC00152 #2 | 5'-AGTTTCAAATTGACATTCCAGACAA-3' | Exon 1 |
| siSTAT3 | 5'-TTTGTTGACGGGTCTGAAGTTGAGA-3' | Exon 9 |

## **Supplementary Table 4**

**Supplementary Table 4: ViewRNA^TM^ probes used for in-situ-hybridization.** Probes were designed and provided by the manufacturer Thermo Fisher.

| **Probe set** | **Specification** | **Supplier** |
| --- | --- | --- |
| LINC00152 exon 1+2 | #VA1-16989-01 | affymetrix |
| LINC00152 exon 3 | #VA6-18301-01 | affymetrix |
| MALAT-1 | #VA6-14180-01 | affymetrix |
| GAPDH | #VA1-10119-01 | affymetrix |
| lacZ | #VF1-12414-01 | affymetrix |
| dapB | #VF6-10407-01 | affymetrix |

## **Supplementary Table 5**

**Supplementary Table 5: Standard qPCR primers used for qPCR analyses.** Primers were designed using primer3 and ordered by Eurofins Scientific, Luxemburg.

| **Name** | **Sequence 5´-3´** |
| --- | --- |
| LINC00152(_a)-for | 5'-CGTGCCTGTCTTCAGATCTTC-3' |
| LINC00152(_a)-rev | 5'-GGGAATCTTTCAGCTGCATT-3' |
| LINC00152_b-for | 5'-gggaaataaatgactggatggtcgctg-3' |
| LINC00152_b-rev | 5'-GGGGATGAGTCGTGATTTTC-3' |
| MIR4435-1-for | 5'-CGTGCCTGTCTTCAGATCTTC-3' |
| MIR4435-1-rev | 5ˈ-CACCAGCATCTTTTCCAACC-3 |
| STAT3-for | 5'-CTGGCCTTTGGTGTTGAAAT-3' |
| STAT3-rev | 5'-AAGGCACCCACAGAAACAAC-3' |
| TRAF3IP2-for | 5'-TATGATTCCCAGCCCCAGGA-3' |
| TRAF3IP2-rev | 5'-AGGGTTGCTAGGGGGTCTAG-3' |
| KRT17P1-for | 5'-AGAACCTCAATGACCGCCTG-3' |
| KRT17P1-rev | 5'-TTGCGGTTCTTCTCTGCCAT-3' |
| TMEM163-for | 5'-GAAACCTCACGAAGCCCAGA-3' |
| TMEM163-rev | 5'-CCGATCAGAACGCCTATGCT-3' |
| EVA1A-for | 5'-GCAACATCCTAGCGGCCTAT-3' |
| EVA1A-rev | 5'-GTAGCGATTCAGGCTCCTGG-3' |
| KRT17P2-for | 5'-AGAACCTCAATGACCGCCTG-3' |
| KRT17P2-rev | 5'-CAGCTCACTGTTGGTAGCCA-3' |
| JADE2-for | 5'-AATGAGCCCACATCTGAGCC-3' |
| JADE2-rev | 5'-CAGCAGTTCCTCTGACAGCA-3' |
| SYNJ2-for | 5'-CACTGCCAACTCCTGTTTGC-3' |
| SYNJ2-for | 5'-CCTGGGCATTGGTGGTTTTG-3' |
| LINC00511-for | 5'-CTAACTCACACCCCACCGAC-3' |
| LINC00511-rev | 5'-AGGGTCTCTCGCTCTTTTGC-3' |
| LAMA4-for | 5'-AGCGGAACACAACAGGAGAG-3' |
| LAMA4-rev | 5'-GCAGGTCATCAGTCAGGTCC-3' |
| DAND5-for | 5'-ATCCACTCTTCTGTGCCTGC-3' |
| DAND5-rev | 5'-CTTTTGGGCTGCAGTGACAC-3' |
| AGTRAP-for | 5'-TGCCTGCTGTGAACCTGAAG-3' |
| AGTRAP-rev | 5'-TGTGGTAGACGAAGCAGCAG-3' |
| LINC01095-for | 5'-AGGAGGCCTGTGATAAATGGG-3' |
| LINC01095-rev | 5'-AGCCAAAAGTAGCACAACAAGC-3' |
| Lnc-POLG-1-for | 5'-CTTGAACCTGGGAGGCAGAG-3' |
| Lnc-POLG-1-rev | 5'-CAGAAGCCAGGGAAGGATGG-3' |
| NFIA-for | 5'-ATCTGAAAGTCCCAGCCAGC-3' |
| NFIA-rev | 5'-GGGTGTGAGAAGTAAGGCCC-3' |
| FLJ32169 fis-for | 5'-CATGGCAAGAGGGAGCAAGA-3' |
| FLJ32169 fis-rev | 5'-AACTTGCCACGATCTCCCAG-3' |
| ATP9A-for | 5'-GATGCTGAACCTGGCTCTGT-3' |
| ATP9A-rev | 5'-AGTGTCTGAGAAGCACGGTG-3' |
| FoxM1-for | 5'-TAAGAGATCCCCTGCCCAAC-3' |
| FoxM-rev | 5'-GGATGAATTTGTTGGGCCCA-3' |
| MAFB-for | 5'-GTGTGCCCCAAGACAAAGTT-3' |
| MAFB-rev | 5'-GGGTTTCAGTCCCAGCTGTA-3' |
| SAA1-for | 5'-AGCCAATTACATCGGCTCAG-3' |
| SAA1-rev | 5'-CACCATGGCCAAAGAATCTC-3' |
| CCNA1-for | 5'-CATGAAGAAGCAGCCAGACA-3' |
| CCNA1-rev | 5'-AGAAACTGGTTGGTGGTTGG-3' |
| FILIP1L-for | 5'-AAACGCCTCCATAACACCAG-3' |
| FILIP1L-rev | 5'-AACCAGTCACAGCCAAAACC-3' |
| FRAS1-for | 5'-AACCTCGTGGGATACTGTGC-3' |
| FRAS1-ref | 5'-TGTCACATGAGGAGCAGGAG-3' |
| MMP2-for | 5'-ATGACAGCTGCACCACTGAG-3' |
| MMP2-rev | 5'-ATTTGTTGCCCAGGAAAGTG-3' |
| U6-for | 5'-CTCGCTTCGGCAGCACA-3' |
| U6-rev | 5'-AACGCTTCACGAATTTGCGT-3' |

## **Supplementary Table 6**

**Supplementary Table 6: ChIRP DNA oligos used for RNA pulldown.** The 3ˈ-biotinylated DNA oligonucleotides (= BITEG; with a TEG spacer between the oligo and biotin) were designed using [www.singlemoleculefish.com](http://www.singlemoleculefish.com) and provided by Eurofins Scientific.

| **target** | **Oligo** | **Sequence** |
| --- | --- | --- |
| lacZ control oligos | lacZ1 | 5'-ccagctttcatcaacattaaatgtg-3'-BITEG |
|  | lacZ2 | 5'-ataaagaaactgttacccgtaggta-3'-BITEG |
|  | lacZ3 | 5'-cgttaaagttgttctgcttcatcag-3'-BITEG |
|  | lacZ4 | 5'-gccgttttcatcatatttaatcagc-3'-BITEG |
|  | lacZ5 | 5'-agttcaatcaactgtttaccttgtg-3'-BITEG |
|  | lacZ6 | 5'-tgataaataaggttttcccctgatg-3'-BITEG |
| LINC00152 oligos | LINC00152.1 | 5'-ttctcattggaacgagatgactcat-3'-BITEG |
|  | LINC00152.2 | 5'-tccattcccaatgatgtacacacga-3'-BITEG |
|  | LINC00152.3 | 5'-tcaggcaccgcttgtctggaatgtc-3'-BITEG |
|  | LINC00152.4 | 5'-gaactgtgctgtgaagatctgaaga-3'-BITEG |
|  | LINC00152.5 | 5'-cctgtttcatctcccagttattcag-3'-BITEG |
|  | LINC00152.6 | 5'-atattcgatcaagtgtgtcatagag-3'-BITEG |
|  | LINC00152.7 | 5'-tcagctgcattccggctgtgatcgg-3'-BITEG |
|  | LINC00152.8 | 5'-ggttggaaccaggccccagggaatc-3'-BITEG |
|  | LINC00152.9 | 5'-caaatgcagaggcctcagagtccac-3'-BITEG |
|  | LINC00152.10 | 5'-catgaccaaaatatcacaggcagac-3'-BITEG |
|  | LINC00152.11 | 5'-cagacaaatgggaaaccgaccagac-3'-BITEG |
|  | LINC00152.12 | 5'- aatgaaggacaagggattaagacac-3'-BITEG |

## **Supplementary Table 7**

**Supplementary Table 7: Primary and secondary antibodies used for Immunoblot detection.**

| **Primary antibodies** | **Note** |
| --- | --- |
| anti-STAT3 | Mouse, monoclonal, 1:1,000 in 5% BSA in TBS-N, Transduction Laboratories, Lexington, Kentucky, USA (S21320) |
| anti-beta-actin | Mouse, monoclonal, 1:1,000 in 5% BSA in TBS-N, Sigma-Aldrich, Taufkirchen, Germany (Clone AC-74) |
| **Secondary antibodies** | **Note** |
| Rabbit-anti-mouse-HRP | 1:10,000 in TBS-N, DAKO, Jena, Germany |

# **Supplementary Figures**

## **Supplementary Figure 1**


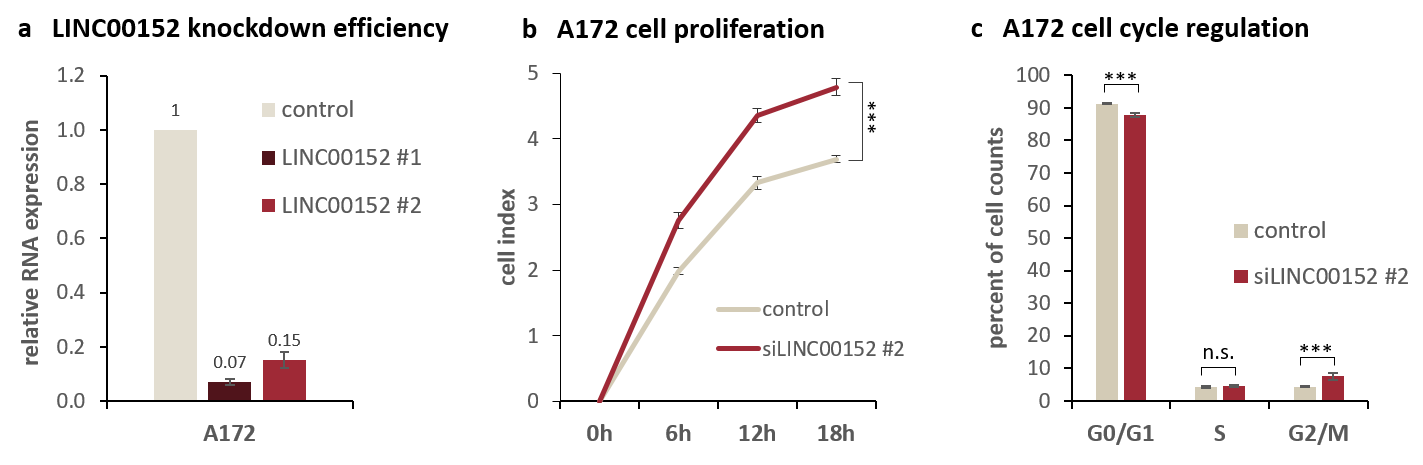


**Supplementary Figure 1: LINC00152 knockdown in A172 glioblastoma cells.** (a) Knockdown efficiencies of siLINC00152 #1 and #2 in A172. RNA was prepared and reverse transcribed 48 h after transfection and knockdown efficiencies were determined by qPCR using specific primers. Values were normalized to U6 expression (housekeeper) and compared to the negative control knockdown. (b) LINC00152 was knocked down with another siRNA targeting the first exon (siLINC00152 #2) and cell proliferation was monitored over time in an incubator equipped with a microscope and camera (XCelligence®). The cell index is a measure of cell proliferation. (c) DNA of A172 cells was labeled with propidium iodide 72 h after siLINC00152 #2 and control siRNA transfection and the DNA content was measured by flow cytometry.

## **Supplementary Figure 2**


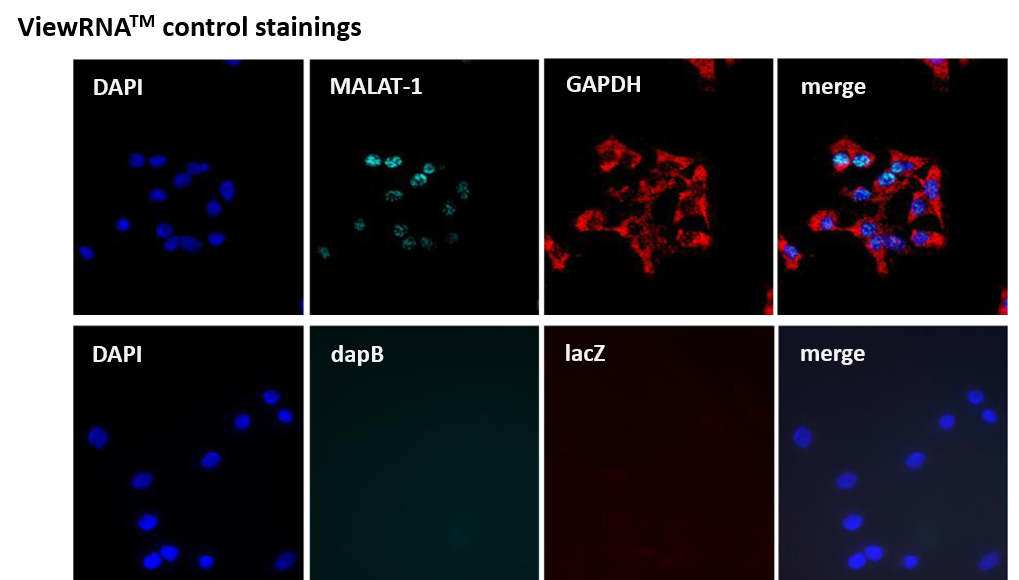


**Supplementary Figure 2: ViewRNA^TM^ control stainings.** The ViewRNA^TM^ staining was performed using the ViewRNA^TM^ ISH Cell Assay Kit according to the manufacturer's instructions. As positive controls the cytoplasmic GAPDH mRNA and the nuclear ncRNA MALAT-1 were used. The bacterial RNAs of lacZ and dapB served as negative controls. After *in situ* hybridization, the nuclei were counterstained with DAPI and images were merged. The fluorescence signals were analyzed microscopically using FITC (488 nm), Cy3 (550 nm) and Cy5 (650 nm) filters.

## **Supplementary Figure 3**


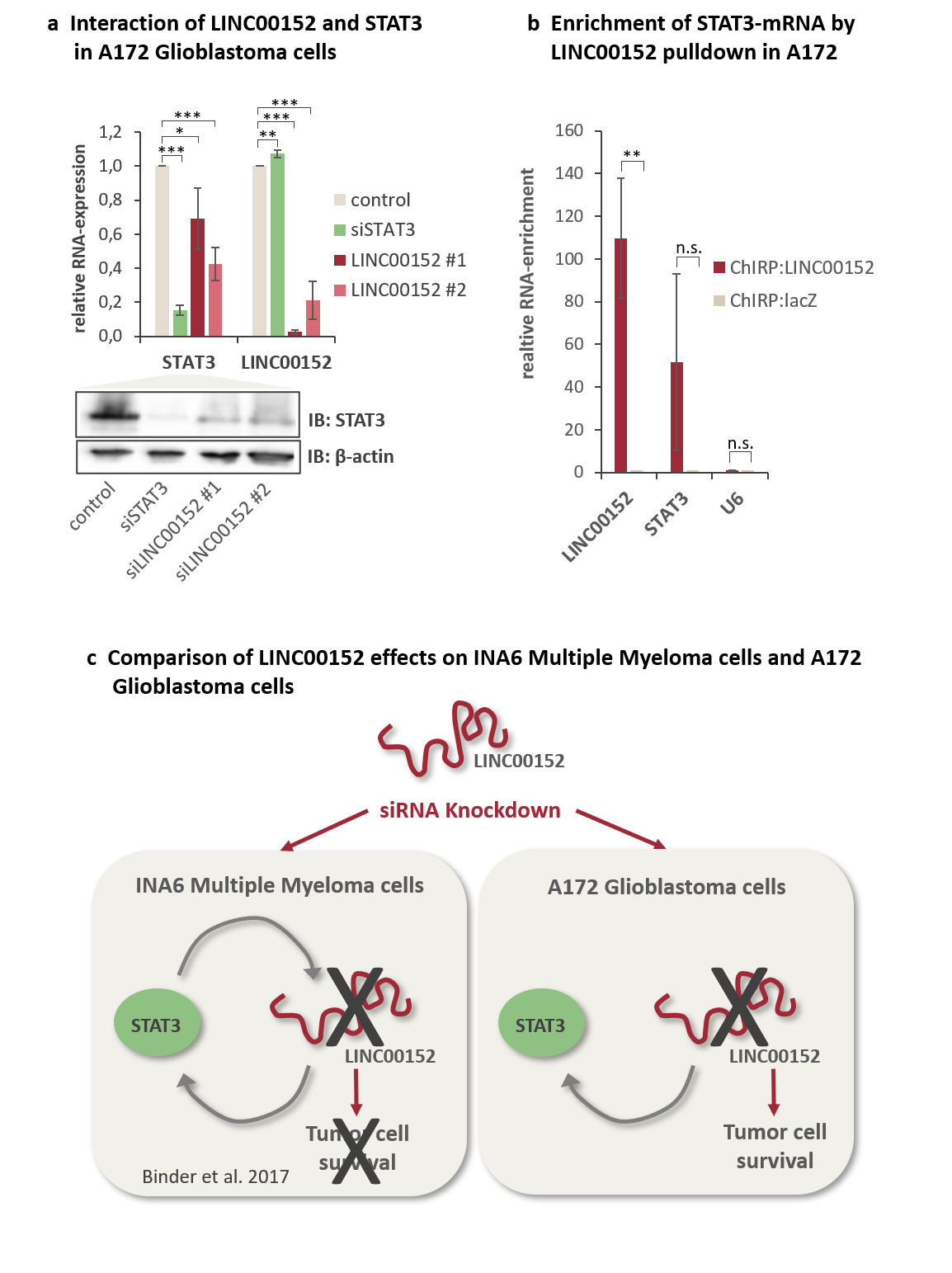


**Supplementary Figure 3: Interaction of LINC00152 and STAT3.** (a) STAT3 mRNA and protein levels depend on LINC00152 in A172 GBM cells. Knockdowns of LINC00152 and STAT3 were conducted in A172 cells, followed by RNA- and protein-isolation after 24 h and 48 h, respectively. RNA expression was carried out by qPCR; protein expression by SDS-PAGE and immunoblotting using a specific anti-STAT3-antibody as well as an antibody detecting β-actin as a loading control. (b) Interaction of LINC00152 and STAT3 mRNA was determined by ChIRP using probes targeting LINC00152 in formaldehyde fixed A172 cells. Oligos for bacterial lacZ RNA served as negative controls. Co-precipitated RNAs were isolated, reverse transcribed and STAT3 analyzed by qPCR. STAT3-RNA enrichment was measured using specific primers. The enrichment of LINC00152 was determined as a control for specific binding and the enrichment of U6-RNA as a control for unspecific binding. (c) Schematic representation of LINC00152 function in INA-6 Multiple Myeloma (MM) cells and in A172 Glioblastoma cells. In INA-6 cells, LINC00152 sustains a positive feedback with STAT3 and LINC00152 knockdown leads to a drastic decrease of tumor cell survival, whereas in A172 cells LINC00152 positively regulates STAT3 expression and cell vitality is not affected by LINC00152 knockdown.

**Supplementary Figure 4**


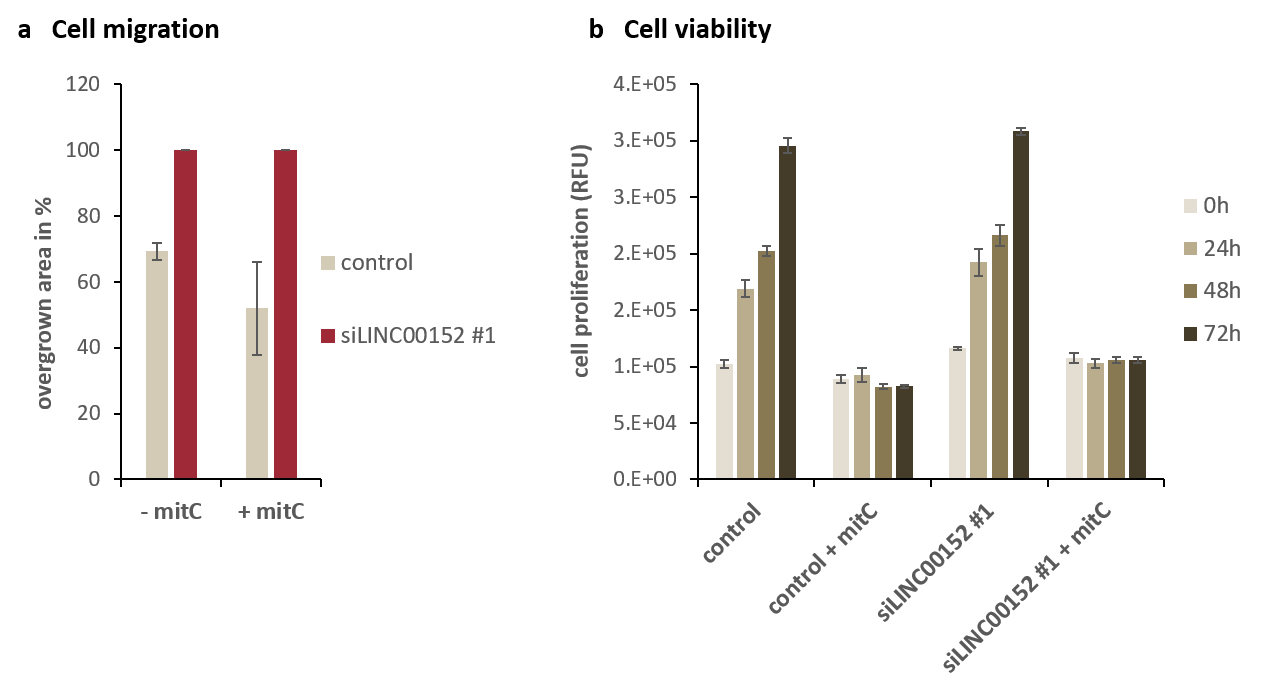


**Supplementary Figure 4: Migration (a) and viability (b) of A172 cells in relation to LINC00152 after mitomycin C incubation.** (a) A172 cells transfected with siLINC00152 #1 or with a negative control siRNA were incubated with 50 μg/ml mitomycin C. After 3 h 1x10^4^ cells were seeded into each chamber of the migration inlay. After 24 h, inlays were removed generating a cell-free gap. The width of the overgrown area was analyzed over the next 24 h microscopically (n=3). The images were analyzed using a Scratch Assay Analyzer (ImageJ <https://imagej.nih.gov/ij/>). (b) A172 cells were transfected as described above, incubated with or without mitomycin C, and 5x10^3^ cells were seeded per well. Cell viability was determined by measuring ATP levels using the CellTiter-Glo® Luminescent Cell Viability Assay (Promega) (n=5). The fluorescence intensity correlates with the number of cells, allowing to draw conclusions on cell proliferation and viability.

## **Supplementary Figure 5**


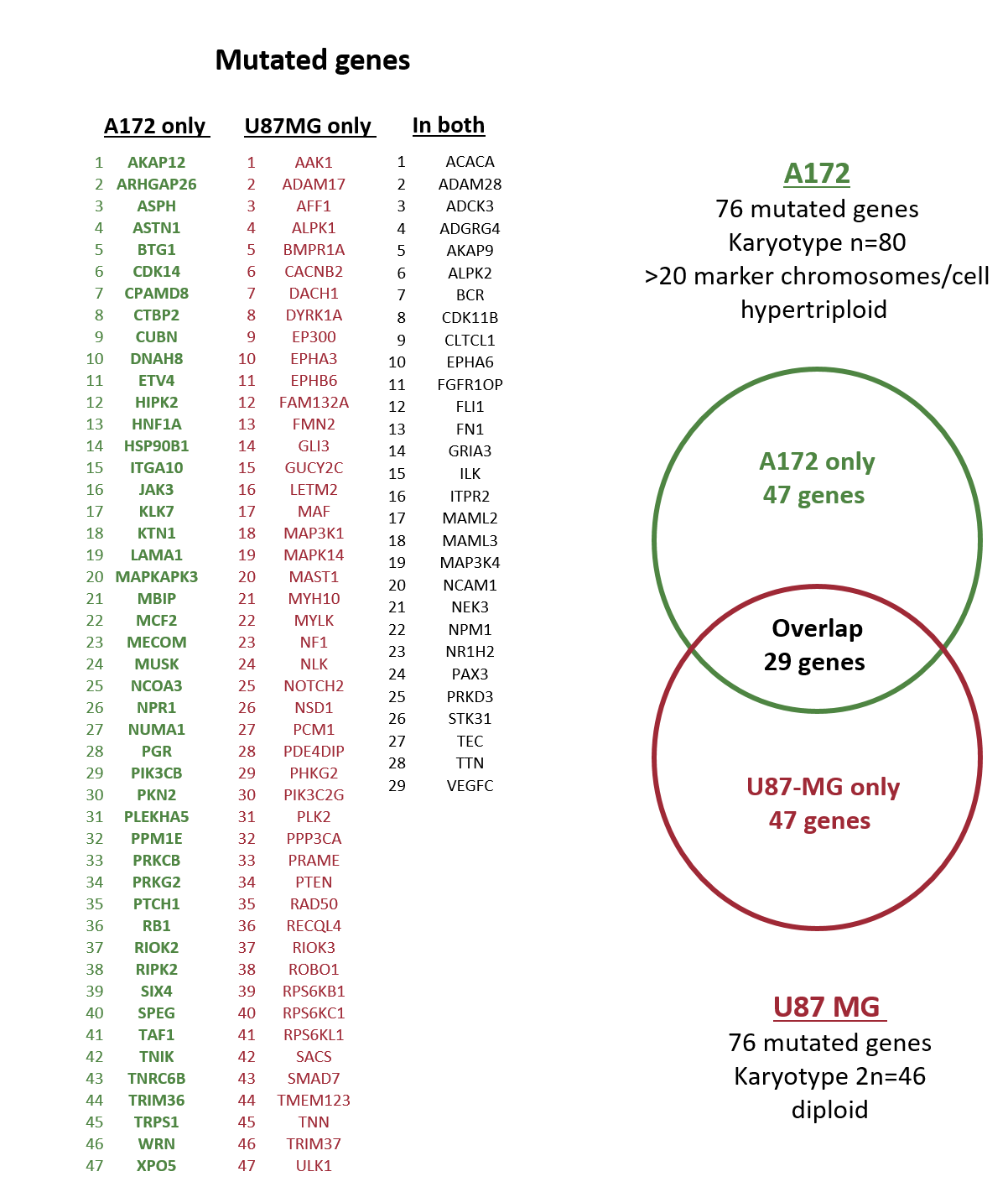


**Supplementary Figure 5:** Mutated genes per cell line were taken from Harmonizome (Rouillard et al., Database (Oxford), 2016) of the Ma'ayan Laboratory of Computational Systems Biology.

## **Supplementary Figure 6**


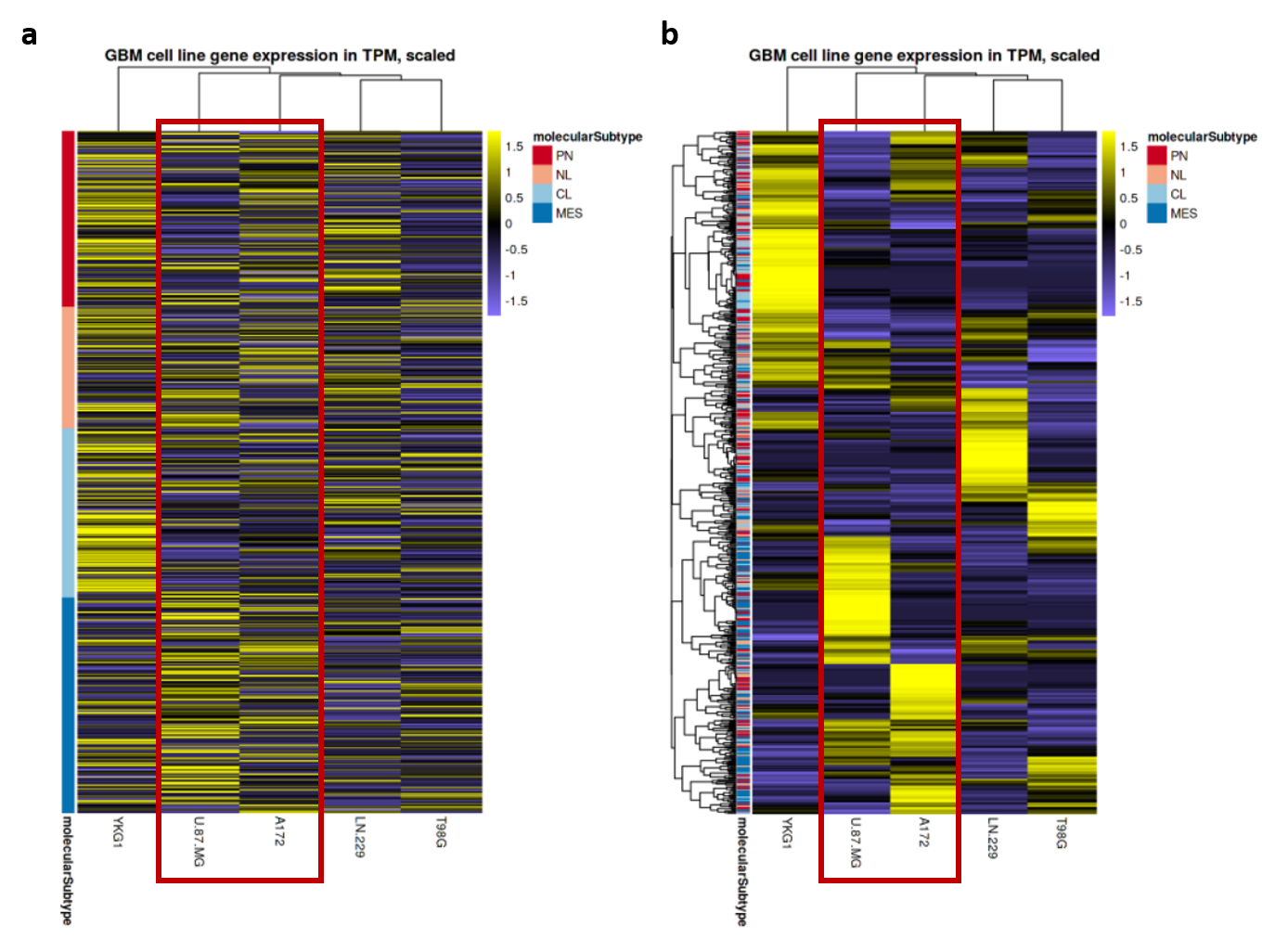


**Supplementary Figure 6:** To assign different cell lines to GBM molecular subgroups, we compared the gene expression from EMBL-EBI Expression Atlas for the GBM cell lines A172, U87-MG, T98G, LN229, and YKG1 ([EMBL-EBI Expression Atlas](https://www.ebi.ac.uk/gxa/experiments/E-MTAB-2770/Results?specific=true&geneQuery=%255B%255D&filterFactors=%257B%2522CELL_LINE%2522%253A%255B%2522A172%2522%252C%2522LN-229%2522%252C%2522T98G%2522%252C%2522U-87%2520MG%2522%252C%2522YKG1%2522%255D%257D&cutoff=%257B%2522value%2522%253A0.5%257D&unit=%2522TPM%2522)) with GBM molecular subgroups described by Verhaak et al. ([840 molecular subgroup defining genes](https://gdc.cancer.gov/about-data/publications/gbm_exp/TCGA_unified_CORE_ClaNC840.txt)). Expression and subgroups were matched via gene symbols and aliases and plotted in a heatmap using R version v4.0.0 (R Core Team, 2020) and pheatmap v1.0.12 (Kolde, 2019). Of the 840 original genes 615 genes with corresponding molecular subgroup and Ensembl Gene ID were included. Heatmap (a) is column wise clustered via Euclidian distances whereas heatmap (b) is clustered column wise and row wise. Both heatmaps are scaled per gene (row wise).

## **Supplementary Figure 7**


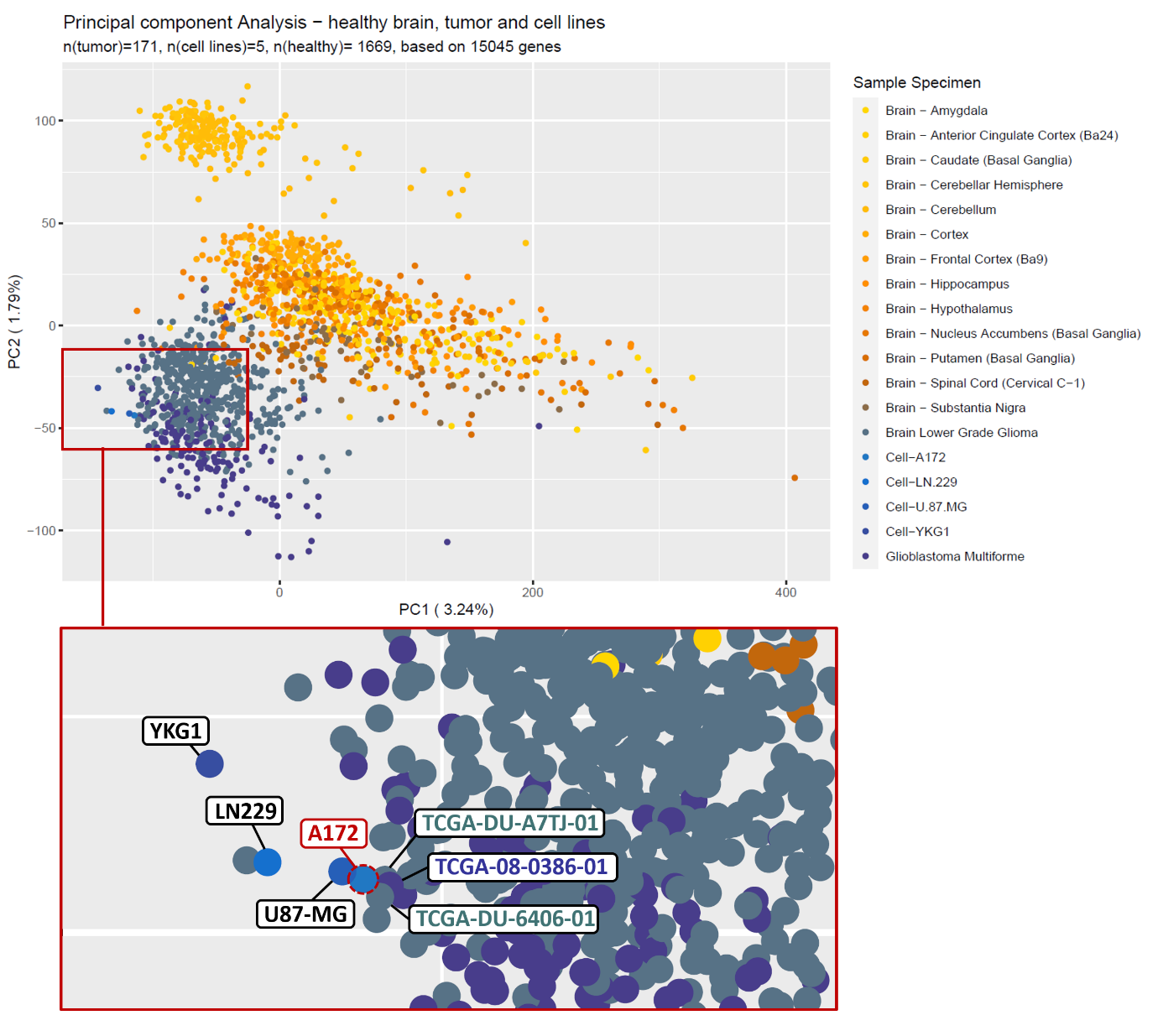


**Supplementary Figure 7:** Principal component analysis based on all genes with expression in all samples. Normalized expression data from the XenaBrowser (TCGA TARGET GTEx gene expression by UCSC TOIL RNA-seq recompute <https://xenabrowser.net/>) was filtered by studies TCGA and GTEx and for primary site “Brain”. Cell line datasets were obtained from EMBL-EBI Expression Atlas <https://www.ebi.ac.uk/gxa/home>. Molecular subgroup defining genes were obtained from the link <https://gdc.cancer.gov/about-data/publications/gbm_exp/TCGA_unified_CORE_ClaNC840.txt> provided by Verhaak et al. 2010. Datasets were filtered to genes expressed in all samples (n_(genes)_=15045). Also, from GTEx dataset only samples processed with Illumina HiSeq 2000, as described in the original dataset, were included in order to keep technical comparability between the samples (Total number of samples = 1845, Number of healthy samples (GTEx) = 1669, Number of cell lines = 5, Number of primary tumor samples (TCGA) = 171). To assess whether genes defining molecular subgroups of GBM the gene set was then subset to all genes of the Verhaak 840 gene subset that were expressed in all samples (n_(genes)_=581). Analyses were performed using R v4.0.0 <https://www.ncbi.nlm.nih.gov/projects/gap/cgi-bin/study.cgi?study_id=phs000424.v8.p2>. PCA was calculated from both data matrices with the R core function prcomp() while the results were plotted with ggplot2 v3.3.5 <https://www.r-project.org/>. Primary healthy brain tissue is displayed in yellow. Primary Low-Grade Glioma in gray, the Glioblastoma cell lines in blue and primary Glioblastoma in dark blue.

## **Supplementary Figure 8**


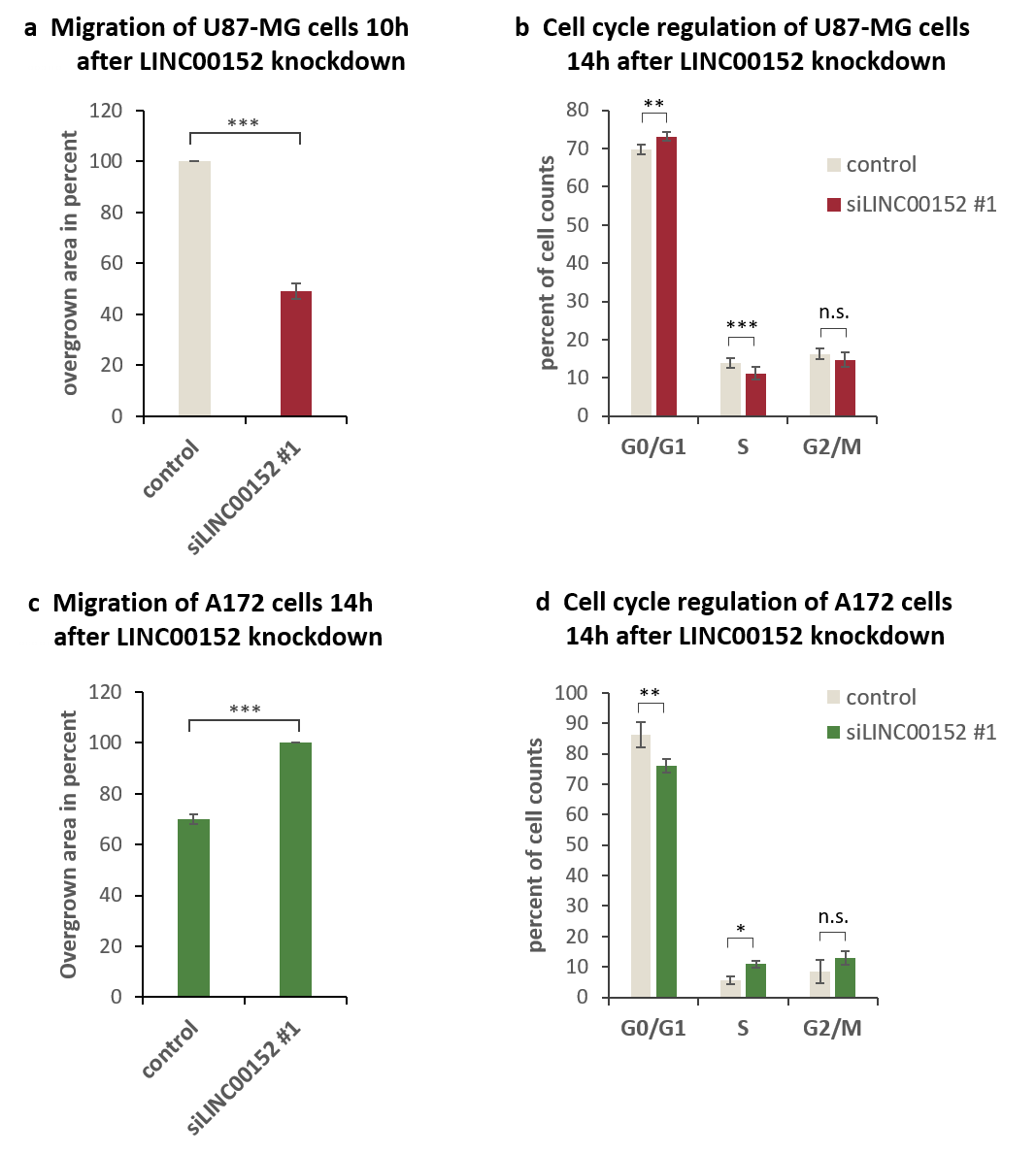


**Supplementary Figure 8: Contradicting LINC00152 phenotypes in different Glioblastoma cell lines.** Knockdown of LINC00152 downregulates migration of U87-MG (a) but upregulates migration of A172 cells (c) indicating a cell-type specific function in GBM cells. Cell migration was analyzed via Incucyte® after LINC00152 knockdown. 24 h after transfection a defined scratch was applied to a confluent cell layer and cell migration was observed for the following 72 h. Shown is the timepoint of overgrowth (100%) of either control or LINC00152 knockdown cells. For cell cycle analysis, 5 × 10^5^ U87-MG (b) and A172 (d) cells were fixed 72 h after LINC00152 knockdown. After labeling DNA with propidium iodide, the DNA contend was measured by flow cytometry.

## **Supplementary Figure 9**

**
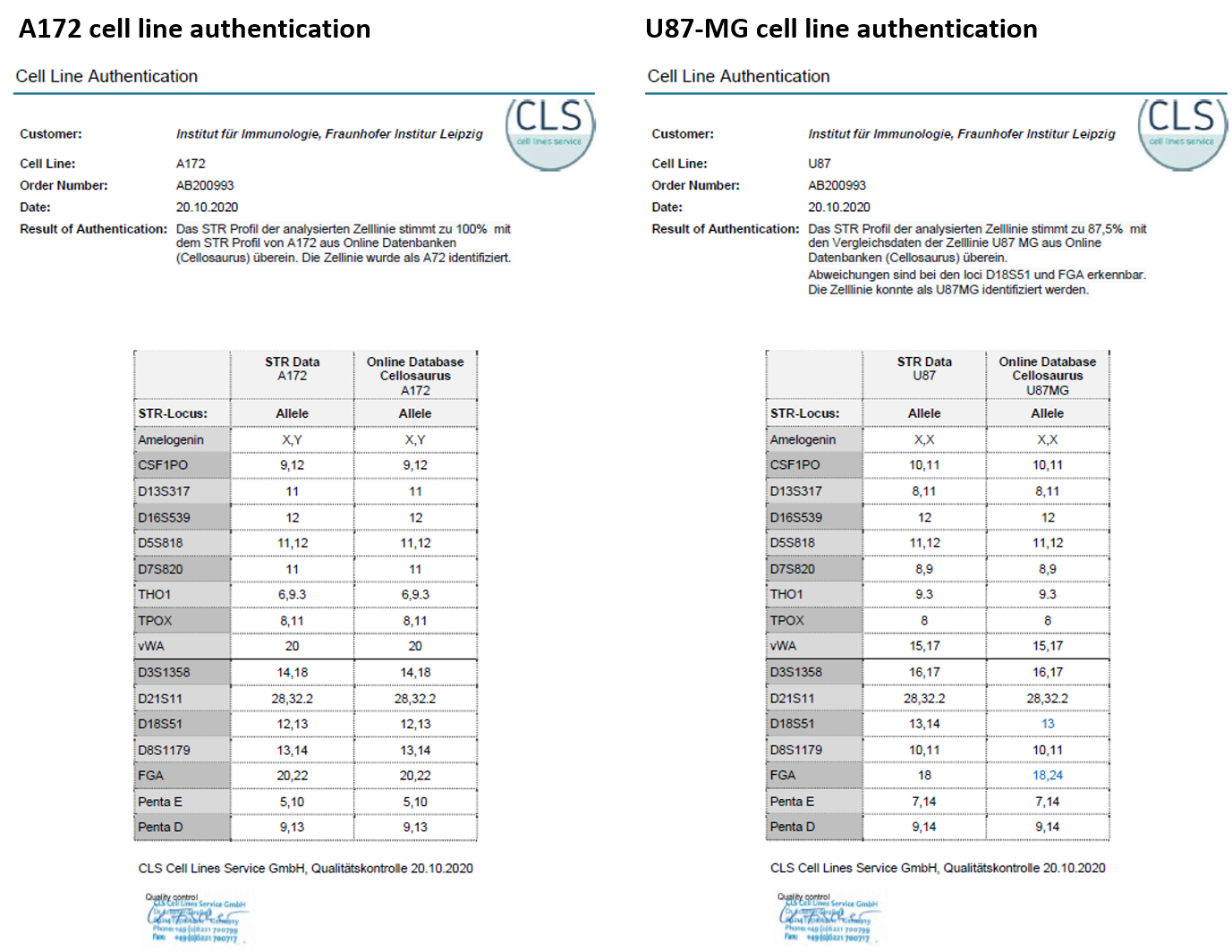
**

**Supplementary Figure 9: Authentication of A172 and U87-MG Glioblastoma cell lines.** Identity of cell lines was determined by the company CLS GmbH (Eppelheim, Germany). The STR profile of A172 agrees to 100% and the STR profile of U87-MG agrees to 87.5% with the STR profiles of each cell line from online databases (Cellosaurus). The cell lines were clearly identified as A172 and U87-MG, respectively.
